# Uncovering the monogenean species diversity of cyprinoid fish in Iraq using an integrative approach

**DOI:** 10.1017/S0031182023001348

**Published:** 2024-02

**Authors:** M. Benovics, C. Rahmouni, E. Řehulková, F. Nejat, A. Šimková

**Affiliations:** 1 Department of Botany and Zoology, Faculty of Science, Masaryk University, Brno, Czech Republic; 2 Department of Zoology, Faculty of Sciences, Comenius University in Bratislava, Bratislava, Slovakia

**Keywords:** Cyprinoidei, *Dactylogyrus*, *Dogielius*, *Gyrodactylus*, Middle East, *Paradiplozoon*, phylogeny, species diversity

## Abstract

The freshwaters of Iraq harbour a high diversity of endemic and phylogenetically unique species. One of the most diversified fish groups in this region is cyprinoids, and although their distribution is relatively well known, their monogenean parasites have only rarely been investigated. Herein, we applied an integrative approach, combining morphology with molecular data, to assess the diversity and phylogeny of cyprinoid-associated monogenean parasites. A total of 33 monogenean species were collected and identified from 13 endemic cyprinoid species. The highest species diversity was recorded for *Dactylogyrus* (Dactylogyridae, 16 species) and *Gyrodactylus* (Gyrodactylidae, 12 species). Four species of *Dactylogyrus* and 12 species of *Gyrodactylus* were identified as new to science and described. Two other genera, *Dogielius* (Dactylogyridae) and *Paradiplozoon* (Diplozoidae), were represented only by 4 and 1 species, respectively. Phylogenetic analyses of the *Dactylogyrus* and *Gyrodactylus* species revealed that the local congeners do not form a monophyletic group and are phylogenetically closely related to species from other regions (i.e. Europe, North Africa and Eastern Asia). These findings support the assumption that the Middle East served as an important historical crossroads for the interchange of fauna between these 3 geographic regions.

## Graphical abstract

**Figure:**
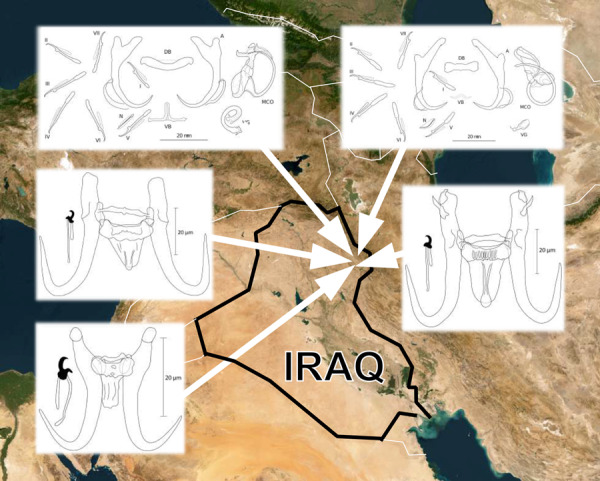

## Introduction

Freshwater ecosystems are home to a remarkable degree of biodiversity (approximately 10% of all so-far-known species, according to Strayer and Dudgeon, 2010) and are undoubtedly one of the most threatened types of ecosystems in the world (Sala *et al*., 2000). In Iraq, freshwater ecosystems provide a variety of services; however, traditional fisheries are quickly being replaced by the farming, cultivation and harvesting of non-native and invasive species. These are, namely, *Carassius auratus* (Linnaeus, 1758), *Ctenopharynodon idella* (Valenciennes, 1844), *Cyprinus carpio* Linnaeus, 1758 and *Hypophthalmichthys molitrix* (Valenciennes, 1844), which pose the greatest threat to the local endemic freshwater fauna and are replacing formerly endemic cyprinids (e.g. Khalaf, 1961; Al-Hassan *et al*., 1989; Jawad, 2006). Currently 6 cyprinoid species (*sensu* Tan and Armbruster, 2018) are recognized as invasive in Iraq (Al-Faisal, 2020), posing a potential threat to the native fauna. The native freshwater fish fauna is relatively well known, according to the most recent checklist by Al-Faisal (2020) and includes 31 species (cyprinoids being the most speciose suborder in the area), out of which more than half are considered as threatened (17 species according to the IUCN Red List, 2023). The relatively high species diversity is mainly due to the geographical position of Iraq, as it overlaps 3 major biodiversity hotspots (Myers *et al*., 2000). The most species-rich genera of cyprinoids are *Luciobarbus*, with 6 species, and *Capoeta* and *Garra*, with 4 each. The freshwater diversity in Iraq is mainly bound to the Euphrates and Tigris basins, as these represent the major river systems in the area. These river systems were important historical dispersion crossroads for cyprinoids and besides the entirely endemic genera (e.g. *Capoeta*), the rivers currently harbour species closely related to the congeners common in Africa (e.g. *Garra*, *Luciobarbus*), eastern Asia (e.g. *Cyprinion*) and Europe (e.g. *Barbus*, *Luciobarbus*) (Kottelat and Freyhof, 2007; Coad, 2010; Yang *et al*., 2015; Froese and Pauly, 2023).

Although the diversity and distribution of cyprinoids have been thoroughly investigated in Iraq (Coad, 2010; Al-Faisal, 2020; Abdullah *et al*., 2022), little is known about their parasites. Such parasites represent a biological threat to already endangered native fish, especially considering the co-invasion of parasites with non-native fish species (Lymbery *et al*., 2014; Benovics *et al*., 2018; Šimková *et al*., 2018; Wilson *et al*., 2019). The highest metazoan parasite diversity in Iraq is reported for monogeneans (e.g. Mhaisen and Al-Rubaie, 2016; Mhaisen and Abdullah, 2017; Mhaisen *et al*., 2019). These ectoparasitic flatworms mainly infest ectothermic vertebrates and several invertebrate taxa, as their life cycle is strictly limited to the aquatic environment. The taxonomy of monogeneans is quite complex and mainly based on the composition and morphology of the hard structures of the attachment organ (i.e. haptor) and the reproductive systems, especially the male copulatory organ (MCO) (Boeger and Kritsky, 1993; Pugachev *et al*., 2009; Řehulková *et al*., 2018). As the localization of a particular species on a host may differ, the haptor is considered as a highly morphologically specialized apparatus; therefore, each host microhabitat (e.g. specific position on the external or internal organ) is associated with a morphological adaptation, i.e. a haptoral morphotype (Rohde, 1979). Cyprinoid fish serve as hosts for several monogenean genera, of which oviparous and gill-infesting *Dactylogyrus* is the most species rich (more than 900 nominal species, according to the latest checklist compiled by Gibson *et al*., 1996). *Dactylogyrus* parasites are almost exclusively associated with cyprinoids and their remarkable species diversity is presumably linked to the diversification and phylogeography of their fish hosts, as each cyprinoid species may potentially serve as a host to at least 1 specialist *Dactylogyrus* species (Ergens, 1970; Šimková and Morand, 2008; Benovics *et al*., 2018). This is especially evidenced in species diversity hotspots or in regions with a high degree of local endemism (e.g. Dupont and Lambert, 1986; El Gharbi *et al*., 1992, 1994; Rahmouni *et al*., 2017; Benovics *et al*., 2018, 2020*b*). Nonetheless, the highest *Dactylogyrus* diversity is harboured by host species with wide distribution ranges (Hoffman, 1999; Seifertová *et al*., 2008; Musilová *et al*., 2009; Molnár, 2012). The other highly diverse genus harboured by cyprinoid fish is viviparous *Gyrodactylus*, whose species are present on fins, skin and gills of their hosts. Currently, more than 400 *Gyrodactylus* species are described around the world (Harris *et al*., 2004), with new species being described almost yearly (e.g. Dos Santos *et al*., 2019; Hansen *et al*., 2020; Truter *et al*., 2022; Shigoley *et al*., 2023). The host specificity in species of *Gyrodactylus* is comparatively high, as in *Dactylogyrus* (more than 70% species infect a single host species; Bakke *et al*., 2002), even in spite of the fact that the life cycle lacks free-living larval stages. However, the taxonomy is usually ambiguous, as the most relevant hard parts are only the haptoral ones. This is because the MCOs are often not well recognized, or not developed (Bakke *et al*., 2002; Huyse and Volckaert, 2005).

The study of the diversity of parasites in Iraq can be traced back to the 1970s, to the work of Herzog (1969), who examined various fishes from markets and identified 4 monogenean species besides endoparasitic helminths. Since then, the vast majority of local research has been conducted only on a small number of targeted fish host species (e.g. Al-Rubaie *et al*., 2007; Hussain, 2008; Bashȇ and Abdullah, 2010; Mohammad, 2016), on specific parasite taxa (e.g. Rahemo, 1980, 1982; Abdullah and Mhaisen, 2005; Al-Ayash *et al*., 2021) or in restricted geopolitical or hydrological regions (e.g. Al-Shaikh *et al*., 1995; Mhaisen, 1995; Muhammad *et al*., 2013; Hashim *et al*., 2015). According to the host–parasite checklists composed for the different Iraq regions (and some additional records; Rasheed and Al-Saadi, 2018), local monogenean fauna comprises more than 107 species belonging to 12 genera (Mhaisen, 1995; Mhaisen and Al-Nasiri, 2012; Mhaisen and Abdullah, 2017; Mhaisen *et al*., 2015, 2019). However, no previous study focusing on the diversity of monogeneans parasitizing fish species in Iraq applied genetic data for taxonomical purposes, nor performed molecular phylogenetic reconstruction. Besides the studies of Koyee and Abdullah (2019) and Benovics *et al*. (2021*a*), no genetic data are so far available for Iraq monogeneans. Recently, an integrative approach combining genetic characterization with commonly used, taxonomically important morphological characters has become the gold standard with respect to taxonomical research on monogeneans (e.g. Řehulková *et al*., 2020; Acosta *et al*., 2022; Bahanak *et al*., 2022; Jin *et al*., 2022; Nitta, 2023), as, only by evaluating both morphological and molecular data, can the potential complexes of cryptic species (morphologically indistinguishable and closely related) be revealed, as previously documented for *Gyrodactylus* (Bueno-Silva *et al*., 2011), *Cichlidogyrus* (Kmentová *et al*., 2016) and *Dactylogyrus* (Rahmouni *et al*., 2017; Benovics *et al*., 2018).

Therefore, the present study aimed to employ for the first time such an integrative approach to investigate the species diversity of monogeneans of cyprinoids in Iraq. The newly obtained molecular data for previously and newly described *Dactylogyrus* and *Gyrodactylus* species were used to investigate the phylogenetic relationships of endemic parasite taxa to congeners from other geographical regions in order to assess the possible phylogeographical scenarios for cyprinoid hosts and their associated monogeneans. Since for freshwater fauna the Middle East served as a dispersion crossroads between 3 continents (Africa, Asia and Europe), we expected that cyprinoid fish would harbour host-specific parasites phylogenetically associated with congeneric species from all these regions.

## Materials and methods

### Collection and identification of fish hosts

**Figure 1.**
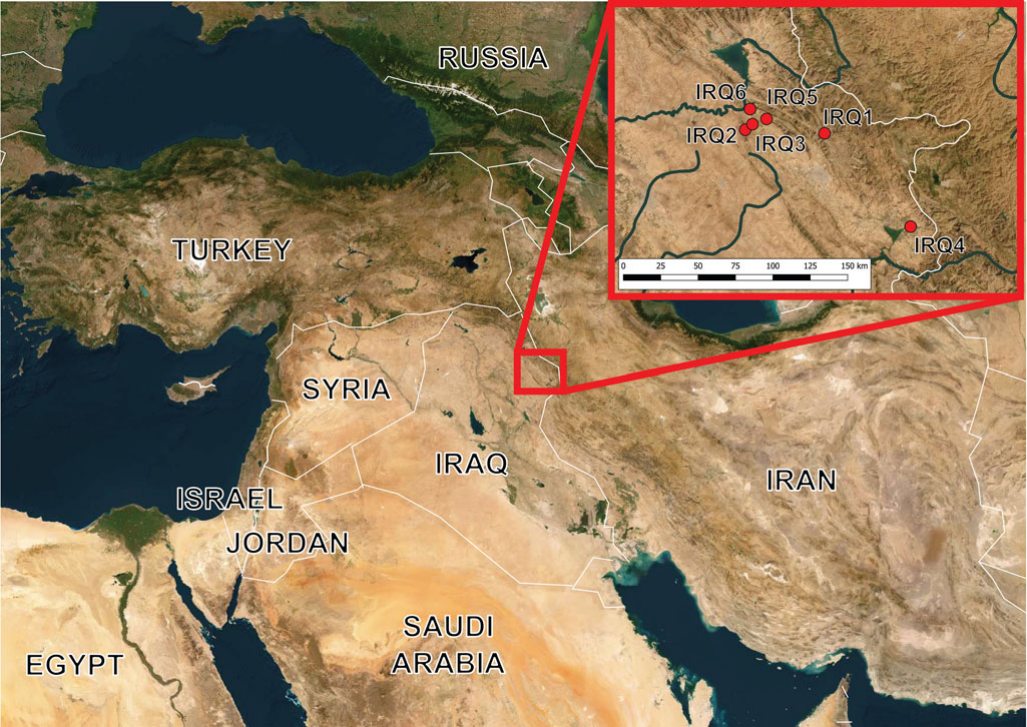
Map with points showing collection sites in Iraq. The codes at points correspond to locality IDs in Table 1.

In September 2021, 13 endemic cyprinoid species were surveyed in Iraq for the presence of ectoparasitic monogeneans. A total of 149 fish specimens were collected at 6 localities in northern and north-western Iraq (see Fig. 1, and also Table 1 for coordinates). The number of collected and examined species represented 41% of indigenous cyprinoid taxa according to the recent checklist compiled by Al-Faisal (2020). The identification of fish was performed by experienced ichthyologists, and the complete cytochrome *b* (cyt-*b*) was genotyped to confirm species assignment following polymerase chain reaction (PCR) protocols published by Viñuela-Rodríguez *et al*. (2021) (see supplementary Table 1 for GenBank accession numbers).

**Table 1.** List of examined cyprinoid species and collected monogenean parasites with the prevalence of individual parasite species in a population of host

Host species	Locality	Coordinates	Locality ID	*N*	*Dactylogyrus acinacus*	*Dactylogyrus barbuli*	*Dactylogyrus carassobarbi*	*Dactylogyrus deziensis*	*Dactylogyrus deziensioides*	*Dactylogyrus goktschaicus*	*Dactylogyrus holciki*	*Dactylogyrus lenkorani*	*Dactylogyrus macrostomi*	*Dactylogyrus microcirrus*	*Dactylogyrus persis*	*Dactylogyrus vistulae*	*Dactylogyrus anoigeus* n. sp.	*Dactylogyrus medicus* n. sp.	*Dactylogyrus regius* n. sp.	*Dactylogyrus rivalis* n. sp.
*Acanthobrama marmid*	Du Choman, Aw-e Shiler River	35°45′49″N 45°27′12″E	IRQ1	10	–	–	–	–	–	–	–	–	–	–	–	–	70	–	–	–
*Alburnus sellal*	wadi Kalat Shirah, tributary of Tabin River	35°47′03″N 44°58′43″E	IRQ2	12	–	–	–	–	–	–	–	–	–	–	–	–	–	–	–	–
	Zahrzi, Tabin River	35°48′32″N 45°01′20″E	IRQ3	7	–	–	–	–	–	–	43	–	–	–	–	–	–	–	–	–
	Grdi Go, Zalm Stream	35°18′26″N 45°58′18″E	IRQ4	5	–	–	–	–	–	–	100	–	–	–	–	–	–	–	–	–
*Alburnus* sp.	Grdi Go, Zalm Stream	35°18′26″N 45°58′18″E	IRQ4	20	–	–	–	–	–	–	50	–	–	–	–	–	–	–	–	–
*Barbus lacerta*	Kani Shok, tributary of Tabin River	35°50′01″N 45°06′16″E	IRQ5	10	–	–	–	–	–	60	–	–	–	–	–	–	–	–	–	–
*Carasobarbus luteus*	Zahrzi, Tabin River	35°48′32″N 45°01′20″E	IRQ3	8	–	–	88	–	–	–	–	–	–	–	100	–	–	–	–	–
	Grdi Go, Zalm Stream	35°18′26″N 45°58′18″E	IRQ4	2	–	–	100	–	–	–	–	–	–	–	50	–	–	–	–	–
*Paracapoeta trutta*	Kani Shok, tributary of Tabin River	35°50′01″N 45°06′16″E	IRQ5	10	–	–	–	–	–	–	–	–	–	80	–	–	–	–	–	–
*Capoeta umbla*	wadi Kalat Shirah, tributary of Tabin River	35°47′03″N 44°58′43″E	IRQ2	12	–	–	–	–	–	–	–	100	–	–	–	–	–	–	–	–
*Cyprinion macrostomum*	wadi Kalat Shirah, tributary of Tabin River	35°47′03″N 44°58′43″E	IRQ2	11	–	–	–	–	–	–	–	–	82	–	–	–	–	–	–	–
*Garra rufa*	By the road Suleymania-Dukan, Little Zab	35°52′53″N 45°00′20″E	IRQ6	10	80	–	–	–	–	–	–	–	–	–	–	–	–	70	–	–
*Chondrostoma regium*	Du Choman, Aw-e Shiler River	35°45′49″N 45°27′12″E	IRQ1	6	–	–	–	–	–	–	–	–	–	–	–	–	–	–	83	–
*Luciobarbus barbulus*	Du Choman, Aw-e Shiler River	35°45′49″N 45°27′12″E	IRQ1	7	–	86	–	100	100	–	–	–	–	–	–	–	–	–	–	–
*Squalius berak*	Kani Shok, tributary of Tabin River	35°50′01″N 45°06′16″E	IRQ5	10	–	–	–	–	–	–	–	–	–	–	–	–	–	–	–	–
*Squalius lepidus*	Du Choman, Aw-e Shiler River	35°45′49″N 45°27′12″E	IRQ1	9	–	–	–	–	–	–	–	–	–	–	–	78	–	–	–	44

Host species	Locality	Coordinates	Locality ID	*N*	*Gyrodactylus azeezsaeedi* n. sp.	*Gyrodactylus blazeki* n. sp.	*Gyrodactylus iraqemembranatus* n. sp.	*Gyrodactylus jurajdai* n. sp.	*Gyrodactylus mhaiseni* n. sp.	*Gyrodactylus sandai* n. sp.	*Gyrodactylus satanicus* n. sp.	*Gyrodactylus vukicae* n. sp.	*Gyrodactylus* sp. 1	*Gyrodactylus* sp. 2	*Gyrodactylus* sp. 3	*Gyrodactylus* sp. 4	*Dogielius* sp.	*Dogielius mokhayeri*	*Dogielius molnari*	*Dogielius cf. persicus*	*Paradiplozoon homoion*
*Acanthobrama marmid*	Du Choman, Aw-e Shiler River	35°45′49″N 45°27′12″E	IRQ1	10	–	–	–	–	–	–	–	–	–	–	–	–	–	–	–	–	30
*Alburnus sellal*	wadi Kalat Shirah, tributary of Tabin River	35°47′03″N 44°58′43″E	IRQ2	12	–	–	8	–	25	–	–	–	–	–	–	–	–	–	–	–	–
	Zahrzi, Tabin River	35°48′32″N 45°01′20″E	IRQ3	7	–	–	14	–	–	–	–	–	–	–	–	–	–	–	–	–	43
	Grdi Go, Zalm Stream	35°18′26″N 45°58′18″E	IRQ4	5	–	–	–	–	60	–	–	–	–	–	20	–	–	–	–	–	80
*Alburnus* sp.	Grdi Go, Zalm Stream	35°18′26″N 45°58′18″E	IRQ4	20	–	5	–	–	–	–	–	–	–	–	–	–	–	–	–	–	50
*Barbus lacerta*	Kani Shok, tributary of Tabin River	35°50′01″N 45°06′16″E	IRQ5	10	–	–	20	–	–	–	–	–	–	–	–	10	–	–	–	–	70
*Carasobarbus luteus*	Zahrzi, Tabin River	35°48′32″N 45°01′20″E	IRQ3	8	–	–	–	–	–	–	–	–	–	–	–	–	–	–	–	–	–
	Grdi Go, Zalm Stream	35°18′26″N 45°58′18″E	IRQ4	2	–	–	–	–	–	–	–	–	–	–	–	–	–	–	–	50	–
*Paracapoeta trutta*	Kani Shok, tributary of Tabin River	35°50′01″N 45°06′16″E	IRQ5	10	–	–	30	–	–	–	–	–	10	–	–	–	–	30	–	–	10
*Capoeta umbla*	wadi Kalat Shirah, tributary of Tabin River	35°47′03″N 44°58′43″E	IRQ2	12	–	–	–	–	–	50	–	–	–	–	–	–	50	–	–	–	–
*Cyprinion macrostomum*	wadi Kalat Shirah, tributary of Tabin River	35°47′03″N 44°58′43″E	IRQ2	11	–	–	–	–	–	9	–	–	–	–	–	–	–	–	55	–	–
*Garra rufa*	By the road Suleymania-Dukan, Little Zab	35°52′53″N 45°00′20″E	IRQ6	10	–	–	–	–	–	–	70	20	–	–	–	–	–	–	–	–	20
*Chondrostoma regium*	Du Choman, Aw-e Shiler River	35°45′49″N 45°27′12″E	IRQ1	6	–	–	–	50	–	–	–	–	–	–	–	–	–	–	–	–	50
*Luciobarbus barbulus*	Du Choman, Aw-e Shiler River	35°45′49″N 45°27′12″E	IRQ1	7	–	–	–	–	–	–	–	–	–	14	–	–	–	–	–	–	43
*Squalius berak*	Kani Shok, tributary of Tabin River	35°50′01″N 45°06′16″E	IRQ5	10	10	–	–	–	–	–	–	–	–	–	–	–	–	–	–	–	30
*Squalius lepidus*	Du Choman, Aw-e Shiler River	35°45′49″N 45°27′12″E	IRQ1	9	–	–	–	–	–	–	–	–	–	–	–	–	–	–	–	–	33

The prevalence is in %. *N* = number of processed fish specimens per population. Locality IDs correspond to those in Fig. 1.

### Collection, fixation, identification and quantification of monogenean parasites

The body surface (including head cavities), fins and gills of freshly killed fishes were examined under a dissection microscope for the presence of ectoparasitic monogeneans, which were collected using fine needles. Parasite collection and fixation followed Řehulková *et al*. (2018). In short, specimens that were subjected to morphological analysis of the hard structures (i.e. haptoral components and copulatory organs – MCO, vagina) were completely flattened under coverslip pressure and fixed with a mixture of glycerine and ammonium picrate (Malmberg, 1957). For each monogenean species, at least 5 specimens intended for DNA analysis were bisected using fine needles: one-half of the body was fixed in 96% ethanol for DNA extraction; the remaining half (either the posterior part containing the haptoral sclerites of *Gyrodactylus* spp., or the anterior part with the MCO of *Dactylogyrus* spp.) was mounted on a slide for further identification and kept as a hologenophore (*sensu* Pleijel *et al*., 2008). Species identification was performed according to the shape and size of the hard elements, following Pugachev et al. (2009).

Prevalence in the host populations (Table 1), as the percentage of fish infected by a given parasite species, was calculated for each monogenean species, following Bush *et al*. (1997).

### Morphometric data and species description

The mounted monogeneans (or their parts) were studied using an Olympus BX 61 microscope equipped with phase contrast optics. The terminology and measurement procedure for the hard structures adopted here essentially follow those of Malmberg (1970) and Pugachev *et al*. (2009). Measurements of morphometrical characters (in micrometres) were taken using digital image analysis software (StreamMotion, version 1.9.2; Olympus). Meristic data are presented in the tables and are given as means followed by the range in parentheses; the number of specimens measured (in subscript font) is given after the respective parentheses. The dimensions of the body and haptor were obtained from unflattened specimens as the longest body measurements, whereas measurements of the hard structures were taken from completely flattened specimens. Drawings were made with the aid of a drawing attachment and redrawn with a graphics tablet compatible with Adobe Illustrator software. Concerning *Dactylogyrus* species, the numbering of hook pairs (in Roman numerals I–VII) follows Mizelle (1936). Type specimens and hologenophores of the monogeneans studied were deposited in the Helminthological Collection of the Institute of Parasitology of the Czech Academy of Sciences (IPCAS), Czech Republic, under the accession numbers IPCAS M-782–793. To comply with the regulations set out in article 8.5 of the amended 2012 version of the International Code of Zoological Nomenclature (ICZN, 2012), details of the new monogenean species have been submitted to ZooBank.

### DNA extraction and amplification

Prior to DNA extraction, the parasites halves were dried from the ethanol using a vacuum centrifuge. Extraction was performed using DNeasy Blood & Tissue kit (Qiagen, Hilden, Germany) following the standard protocol provided by the manufacturer. For *Dactylogyrus* and *Dogielius*, 2 DNA fragments were amplified. Specifically, a section of the partial *18S* rRNA gene (*18S*) with the complete internal transcribe spacer 1 region (*ITS1*), and the partial 5.8 rRNA gene (*5.8S*) were amplified using either the combination of forward primer S1 and reverse primer IR8 (Šimková *et al*., 2003), or the combination of S1 and reverse primer Lig5.8R if the former combination was not yielding successful amplification (Šimková *et al*., 2003; Blasco-Costa *et al*., 2012). The amplification reactions followed protocols optimized in Benovics *et al*. (2018) and Benovics *et al*. (2020*a*). A DNA fragment of the partial *28S* rRNA gene (*28S*) was amplified using forward primer C1 and reverse primer D2 (Hassouna *et al*., 1984). The amplification reaction for this region followed Benovics *et al*. (2020*a*). For *Gyrodactylus*, the region containing a fragment of *ITS1*, complete *5.8S* rDNA and partial internal transcribe spacer 2 region (*ITS2*) was amplified using the combination of the primers *ITS1*A (forward) and *ITS2* (reverse) (Matějusová *et al*., 2001*a*). The amplification reaction followed the protocol optimized by Kvach *et al*. (2019). For diplozoids, complete *ITS2* was amplified using the forward primer D and reverse primer B1 (Bachellerie and Qu, 1993), and the amplification reaction, including PCR conditions, followed the protocol described in Matějusová *et al*. (2001*b*). The PCR products were checked on 1% agarose gel and subsequently purified using ExoSAP-IT^TM^ (ThermoFisher Scientific, Waltham, MA, USA). Sequencing was performed by Macrogen Europe (Amsterdam, the Netherlands), and was carried out using amplification primers.

### Phylogenetic analyses

Phylogenetic analyses were performed separately for each of 2 highly diversified monogenean taxa (*Dactylogyrus* and *Gyrodactylus*) to infer the relationships of the newly described species to the congeners. The orthologue sequences of congeneric species were aligned using the fast Fourier transform algorithm employing MAFFT (Katoh *et al*., 2002) and applying the G-INS-i refinement method. In instances of concatenated sequence datasets, the alignments were treated as partitioned, and an optimal evolutionary model was selected for each partition individually. The phylogenetic analyses were conducted by means of the maximum likelihood (ML) method and Bayesian inference (BI) in RAxML 8.1.12 (Stamatakis, 2006, 2014) and MrBayes 3.2. (Ronquist *et al*., 2012), respectively. For both analyses, all parameters were *a priori* set free to simulate a general time reversible evolutionary model and without reducing the robustness of heuristic search. This allowed respective algorithms to select the optimal model for DNA evolution over the initial search period. The nodal support in each ML analysis was assessed by simulating 1000 pseudoreplicates. Bayesian analyses were run for 5 000 000 generations, with a tree sampling frequency every 100. After checking that the standard deviation fell under 0.01, the first 30% of samples were discarded as representing an initial burn-in period. The convergence of 2 parallel runs was checked in Tracer 1.7.1. (Rambaut *et al*., 2018). Posterior probabilities for each tree node were calculated as the frequency of samples recovering a given clade. The outgroups for each phylogenetic analysis were selected individually to represent phylogenetically sister taxa.

The sequence dataset for assessing phylogenetic relationships of *Dactylogyrus* spp. was built of concatenated sequences of *18S* and *28S*. Regions containing *ITS1* were omitted from the analyses due to *ITS1's* hypervariability and problematic alignment when comparing phylogenetically divergent taxa (see Benovics *et al*., 2018, 2021*a*, 2023). Orthologue *18S* and *28S* sequences from a total of 97 *Dactylogyrus* species representing all so-far-known (Šimková *et al*., 2022) phylogenetic lineages were retrieved from the GenBank database (see supplementary Table 2 for metadata and GenBank accession numbers). The species *Ancyrocephalus percae* (Ergens 1966) was selected as the outgroup for phylogenetic reconstruction, following Mendoza-Palmero *et al*. (2015).

The alignment for assessing phylogenetic relationships in *Gyrodactylus* was built of orthologue sequences of the region containing partial *ITS1*, complete *5.8S* and partial *ITS2*. Sequences from a total of 38 congeneric species were retrieved from the GenBank database (see supplementary Table 3 for metadata and GenBank accession numbers), and *Macrogyrodactylus karibae* Douëllou and Chishawa, 1995 was selected as outgroup, following Přikrylová *et al*. (2013). The sequences were carefully selected to represent the individual *Gyrodactylus* lineages and to concur with the length of the newly generated sequences from the species collected in this study.

## Results

### Overall diversity of collected monogenean taxa

A total of 33 monogenean species belonging to 4 genera (*Dactylogyrus*, *Dogielius*, *Gyrodactylus* and *Paradiplozoon*) were collected from the fins and gills of the examined cyprinoid hosts. The prevalence of each parasite species is shown in Table 1. Monogenean communities with the greatest species richness were harboured by *Garra rufa* (Heckel, 1843) from the Little Zab River, *Paracapoeta trutta* (Heckel, 1843) from Kani Shok and *Luciobarbus barbulus* (Heckel, 1847) from the Aw-e Shiler River, where 5 monogenean species were reported. In contrast, populations of *Acanthobrama marmid* Heckel, 1843, *Alburnus sellal* Heckel, 1843 (at the Grdi Go collection site), *Alburnus* sp., *Carasobarbus luteus* Heckel, 1843 (from the Tarbin River) and *Squalius berak* harboured only 2 monogenean species each. The most species-diverse genus was *Dactylogyrus* (Dactylogyridae) with 16 species, followed by *Gyrodactylus* (Gyrodactylidae) with 12 species. Only 4 species of *Dogielius* (Dactylogyridae) were recorded on the examined cyprinoids, each from a single endemic host species. *Dogielius molnari* Jalali, 1992 and *D. mokhayeri* Jalali and Molnár, 1990 were collected from *Cyprinion macrostomum* Heckel, 1843 and *P. trutta*, respectively. A potentially new species for science, *Dogielius* sp., was collected from *Capoeta umbla* (Heckel, 1843) at wadi Kalat Shirah. *Dogielius* cf. *persicus* Molnár and Jalali, 1992 was collected from *C. luteus* – however, only at the Grdi Go site, where only 2 *C. luteus* specimens were collected and examined. The last reported monogenean genus was *Paradiplozoon* (Diplozoidae), with a single representative, *Paradiplozoon homoion* Bychowsky and Nagibina, 1959, which was recorded from 10 of the investigated cyprinoid species. The highest prevalence of *P. homoion* was recorded on *A. sellal* at Grdi Go (*P* = 70%). *Dactylogyrus* and *Gyrodactylus* species diversity and phylogeny, and descriptions of the new species are presented in the subsequent sections below.

### Species diversity of *Dactylogyrus* parasites in Iraq

A total of 12 of the 13 investigated cyprinoid species were parasitized by *Dactylogyrus* species. Eight cyprinoid species were parasitized by a single *Dactylogyrus* species, whilst the remaining 4 cyprinoid species were parasitized by 2 or 3 *Dactylogyrus* species. A total of 16 *Dactylogyrus* species were identified. The majority of *Dactylogyrus* species were recorded only on a single host species. Only *Dactylogyrus holciki* Molnár and Jalali, 1992 was collected from 2 congeneric hosts – *A. sellal* and *Alburnus* sp. The prevalence of *D. holciki* differed between 2 populations of *A. sellal*. Nonetheless, its prevalence also differed between 2 *Alburnus* species from the same locality (Table 1). The highest number of *Dactylogyrus* species (3) were recorded on *L. barbulus* at Du-Choman (the Aw-e Shiler River).

Four new *Dactylogyrus* species were found on the gills of endemic cyprinoids and are described below. Each of them was collected from only a single host species (i.e. *A. marmid*, *Chondrostoma regium* (Heckel, 1843), *G. rufa* and *Squalius lepidus* Heckel, 1843). Except for *Dactylogyrus medicus* n. sp. from *G. rufa*, all other new species were obtained from the same site on the Aw-e Shiler River.

### Phylogenetic relationships of *Dactylogyrus* in Iraq

**Figure 2.**
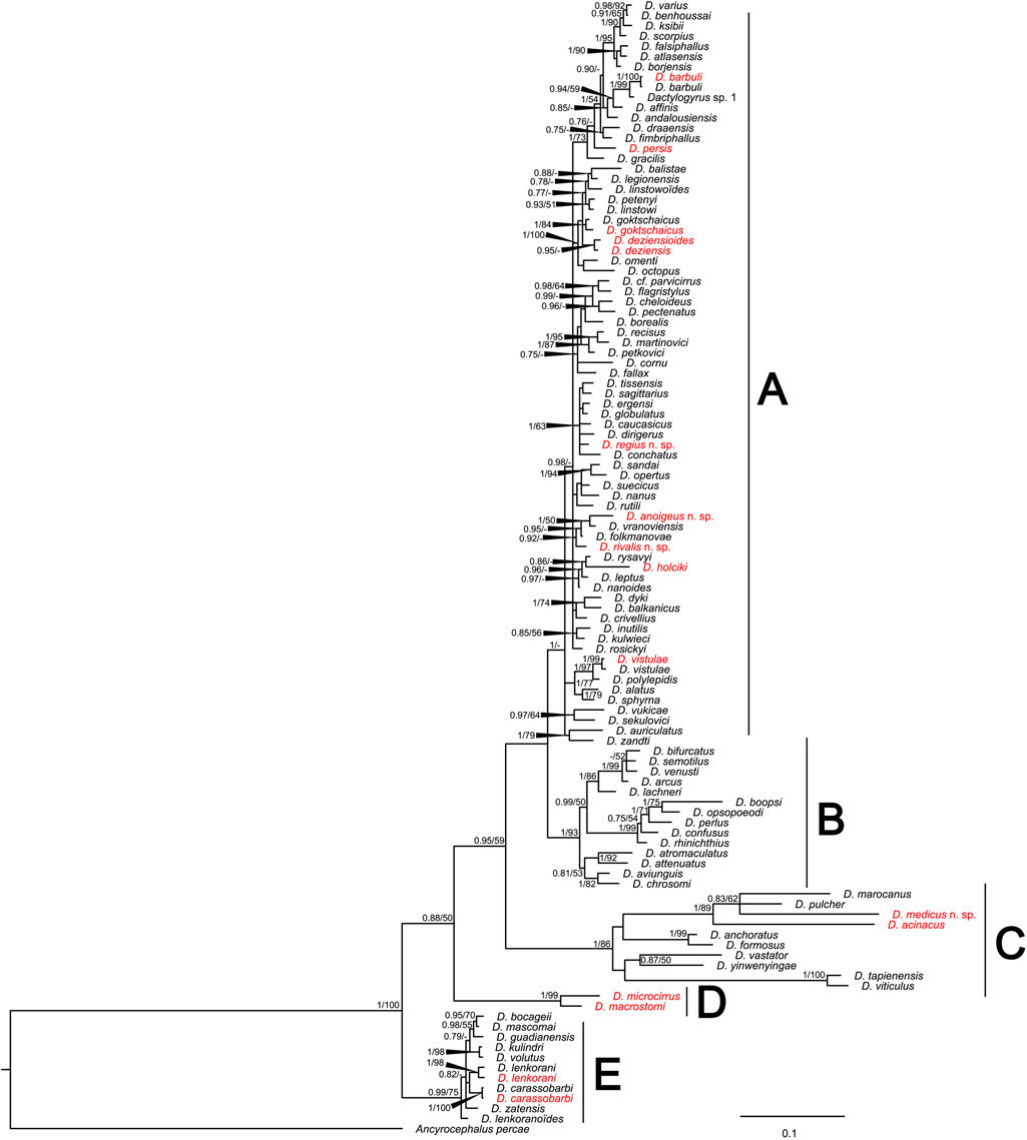
Phylogenetic tree of 105 *Dactylogyrus* spp. parasitizing various cyprinoid fish hosts. The tree is based on 111 combined sequences of partial genes coding *18S* and *28S* rRNA, and rooted using *Ancyrocephalus percae*. Values at the nodes indicate posterior probabilities from BI and bootstrap values from ML analyses. Dashes indicate values below 0.70 and 50, respectively. Letters (A–E) represent specific well-supported clades. The newly described and newly reported species from this study are in red.

The final concatenated nucleotide alignment comprising partial *18S* and *28S* included 110 sequences of 105 *Dactylogyrus* species (4 previously published conspecific sequences were used to confirm the identity of newly collected species) and spanned 1148 unambiguously aligned nucleotide positions (429 bp for *18S*; 719 bp for *28S*). Both phylogenetic analyses (BI and ML) generated trees with identical topologies and differed only in their nodal support (see tree generated by BI in Fig. 2).

The phylogenetic reconstruction divided all *Dactylogyrus* species into 5 well-supported lineages. Lineage A included 10 *Dactylogyrus* species collected in this study, which were in paraphyly. Our results suggest that 4 North American *Dactylogyrus* species (i.e. *D. parvicirrus* Seamster, 1948, *D. flagristylus* Chien, 1974, *D. cheloideus* Rogers, 1967 and *D. pectenatus* Mayes, 1977) form a nested group within lineage A; however, the position of this group to other congeners within lineage A was not well resolved. *Dactylogyrus anoigeus* n. sp. from *A. marmid*, and *D. rivalis* n. sp. from *S. lepidus* were revealed to be phylogenetically close to *D. folkmanovae* Ergens, 1956 and *D. vranoviensis* Ergens, 1956, both common species of *Squalius* spp. in Europe and the Middle East. *Dactylogyrus regius* n. sp., described from *C. regium*, grouped with common species of *Chondrostoma* and *Parachondrostoma* (the *Chondrostoma sensu lato* group) in Europe and the Middle East, namely *D. ergensi* Molnár, 1964, *D. dirigerus* Gussev, 1966, *D. conchatus* Benovics, Francová, Volta, Dlapka and Šimková, 2021 and *D. globulatus* Benovics, Francová, Volta, Dlapka and Šimková, 2021. All these species share remarkable similarities in their hard taxonomically important characters with *D. sagittarius* Benovics, Francová, Volta, Dlapka and Šimková, 2021, *D. caucasicus* Mikailov and Shaova, 1973, *D. rutili* Glaser, 1965 and *D. tissensis* Zachvatkin, 1951. Lineage B included all other analysed North American *Dactylogyrus* species. Lineage C included *Dactylogyrus* species originating in eastern and southeastern Asia, associated with *C. carpio* and *Carassius* spp. fishes, Indonesia (i.e. *D. tapienensis* Chinabut and Lim, 1993 and *D. viticulus* Chinabut and Lim, 1993), North Africa (*D. marocanus* El Gharbi, Birgi and Lambert, 1994) and the Middle East (i.e. *D. acinacus* Gussev, Jalali and Molnár, 1993, *D. pulcher* Bychowsky, 1957 and the newly described *D. medicus* n. sp. from *G. rufa*). Lineage D included only 2 species collected in Iraq – specifically, *D. microcirrus* Gussev, Jalali and Molnár, 1993, originally described from *C. trutta* (syn *Paracapoeta trutta* [Heckel, 1843]) in Iran, and *D. macrostomi* Gussev, Ali, Abdul-Ameer, Amin and Molnár, 1993, described from *C. macrostomum*, also in Iran. Finally, lineage E encompassed species parasitizing cyprinoids in the western peri-Mediterranean region and 2 Middle Eastern species, which were present also in Iraq (i.e. *D. carassobarbi* Gussev, Jalali and Molnár, 1993 and *D. lenkorani* Mikailov, 1974).

### Morphological and molecular characterization of the new *Dactylogyrus* species

#### ***Dactylogyrus anoigeus*** **Řehulková n. sp.** (**Fig. 3**)

**Type-host:** *Acanthobrama marmid* Heckel, 1843 (Cyprinoidei: Leuciscidae).

**Figure 3.**
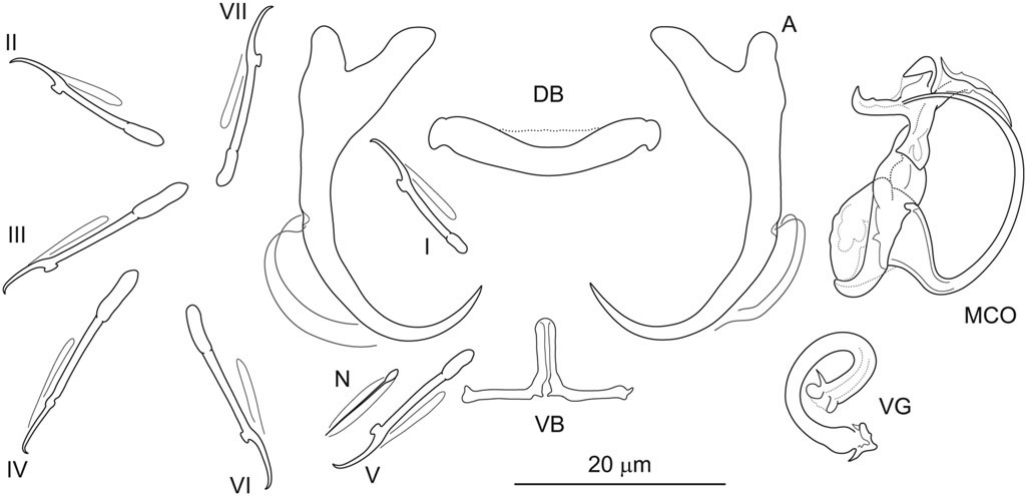
Hard structures of *Dactylogyrus anoigeus* n. sp. ex *Acanthobrama marmid*. A, anchor; DB, dorsal bar; VB, ventral bar; N, needle; I–VII, hooks; VG, vagina; MCO, male copulatory organ.

**Type-locality:** Du Choman, the Aw-e Shiler River, Sulaymaniyah Province, Iraq.

**Site on host:** Gill filaments.

**Type-material:** Holotype, 2 paratypes, 4 hologenophores (IPCAS M-790).

**Representative DNA sequence:** A nucleotide sequence of the partial gene for *28S* rRNA (733 bp long; OR817682), and nucleotide sequences representing a fragment (955 bp long; OR817699) including the partial gene for *18S* rRNA (488 bp), and the *ITS1* region (467 bp). No intraspecific variability was found.

**Infection indices:** Prevalence 70%, 1–4 monogeneans per infected host.

**Etymology:** The specific name is from Greek (*anoigeus* = opener) and refers to the shape of the distal part of the accessory piece of the MCO.

**ZooBank registration (LSID):** urn:lsid:zoobank.org:act:EB687773-3B01-4741-B6D0-02CDFE4DF023.

**Description:** (Dimensions of the hard structures are given in Table 2.) Two pairs of anchors with roots of similar lengths (inner root slightly longer, with flattened termination), elongate shaft bent near its proximal third, recurved point not well demarcated from the shaft and extending well past level of tip of inner root. Dorsal bar broadly V-shaped, weakly narrowed medially, with anteromedial inconspicuous membrane. Ventral bar vestigial, inverted T-shaped, 3-armed. One pair of needles located near hooks of pair V. Seven pairs of hooks; each with delicate point, truncate thumb and shank inflated along proximal 1/3; filamentous hook (FH) loop extending to near level of shank inflation. MCO composed of basally articulated copulatory tube and accessory piece. Copulatory tube with base angularly demarcated from C-shaped shaft. Accessory piece appearing as a plaited rod encircling partially the base and distally formed as a tin opener guiding the end of the tube. Vagina a curled short tube.

**Table 2.** Morphometric data for newly described *Dactylogyrus* species

*Dactylogyrus* species	*D. anoigeus* n. sp.	*D. medicus* n. sp.	*D. regius* n. sp.	*D. rivalis* n. sp.
Host species	*A. marmid*	*G. rufa*	*C. regium*	*S. lepidus*
Body				
Length	235 (221–243)_3_	259 (248–273)_3_	330 (315–346)_2_	451 (340–515)_5_
Width	35 (29–38)_3_	56 (48–61)_4_	89 (85–92)_2_	90 (71–127)_5_
Haptor				
Length	35 (30–37)_3_	57 (55–60)_4_	52 (49–56)_2_	55 (48–69)_4_
Width	56 (54–59)_3_	89 (85–92)_4_	89 (87–91)_2_	101 (94–131)_4_
Anchors				
Length	28 (27–30)_6_	32 (30–34)_6_	33 (32–35)_4_	36 (35–39)_5_
Main part	26 (24–27)_6_	24 (22–26)_6_	28 (26–30)_4_	32 (31–34)_5_
Inner root	9 (8–10)_6_	13 (13–14)_6_	12 (10–12)_4_	15 (15–16)_5_
Outer root	4 (4–5)_6_	3 (2–3)_6_	4 (3–5)_4_	4 (3–5)_5_
Point	8 (8–9)_6_	8 (7–8)_6_	7 (6–8)_4_	6 (5–8)_5_
Dorsal bar				
Length	5 (5–6)_6_	5 (4–5)_5_	7 (7–8)_3_	7 (6–8)_6_
Width	23 (21–28)_6_	17 (16–18)_6_	22 (21–23)_4_	26 (25–27)_6_
Ventral bar				
Length	8 (8–10)_5_	3 (2–4) _4_	9 (9–10)_3_	11 (10–12)_4_
Width	19 (17–21)_6_	11 (9–12)_4_	19 (18–21)_3_	22 (21–22)_4_
Needle				
Length	11 (10–11)_4_	10 (10–11)_3_	10 (10–11)_2_	11 (10–11)3
Hooks				
Length	19 (15–22)_4_	18 (15–22)_2_	22 (18–25)_1_	23 (18–26)_3_
MCO				
Total length	24 (21–26)_6_	30 (29–30)_5_	32 (30–33)_3_	46 (45–48)_6_
Curved length of tube	42 (39–46)_5_	60 (58–61)_5_	73 (69–76)_2_	44 (40–48)_3_
Vagina				
Curved length of tube	27 (25–29)_4_	11 (10–13)_3_	55 (51–58)_3_	18 (15–19)_4_

The first number represents the mean value and is followed by the range of obtained measurements in brackets. The lower index number represents the number of measured specimens.

**Differential diagnosis:** *Dactylogyrus anoigeus* n. sp. belongs to the group of congeners having an inverted T-shaped ventral bar and an MCO between the ʻnanus' and ʻchondrostomi' types (see Pugachev *et al*., 2009). It most closely resembles *D. folkmanovae*, a parasite of *Squalius cephalus* (Linnaeus, 1758) (Pugachev *et al*., 2009), in the comparative morphology of the ventral bar and MCO. In both species, all 3 processes of the ventral bar are similar in length, and each has the same diameter throughout its length, but in *D. anoigeus* n. sp., the termination of the anterior process is rounded (*vs* flattened in *D. folkmanovae*). The MCO of the 2 species is characterized by a sickle-shaped copulatory tube with a recurved base and an accessory piece with a distal widening formed as 2 parts, of which 1 serves as a guide for the distal termination of the tube (pincer-shaped in *D. anoigeus* n. sp. *vs* finger-shaped, with a subterminal filament in *D. folkmanovae*), while the other is directed backwards along the distal curvature of the tube (filamentous in *D. anoigeus* n. sp. *vs* more robust claw-shaped in *D. folkmanovae*). In addition to the above differences, *D. anoigeus* n. sp. is easily differentiated from *D. folkmanovae* by having anchors with roots of similar size (the inner root is markedly longer than the outer root in *D. folkmanovae*) and a recurved point (*vs* open point in *D. folkmanovae*). The sister relationship between *D. anoigeus* n. sp. and *D. vranoviensis* Ergens, 1956 was supported (albeit weakly by ML) by molecular phylogeny. The 2 species share a similar MCO morphology (i.e. a sickle-shaped copulatory tube and an accessory piece encircling partially the base of the tube in the form of a finger-like process), but they clearly differ in that the new species has anchors with a well-developed point (*vs* markedly reduced point in *D. vranoviensis*) and an inverted T-shaped ventral bar (*vs* vestigial rod-shaped ventral bar in *D. vranoviensis*).

Until now, only 2 species of *Acanthobrama* have been reported as hosts for species of *Dactylogyrus*: *A. terraesanctae* (now *Mirogrex terraesanctae* [Steinitz, 1952]) for *D. acanthobramae* Paperna, 1961, *D. carmeli* Paperna, 1961 and *D. sphyrna* Linstow, 1878 (Paperna, 1961, 1964), and *A. simoni* (now *Pseudobrama simoni* [Bleeker, 1864]) for *D. acanthobramis* Zhang and Ji, 1980 and *D. jiayuensis* Zhang and Ji, 1980 (Zhang and Ji, 1980). Of the 5 *Dactylogyrus* species, *D. anoigeus* n. sp. is most similar to *D. acanthobramae*, as the MCO of the 2 species appears to have some common features. Although Paperna's (1961) drawing of the MCO is confusing and not strongly diagnostic, the copulatory tube is depicted and described as ‘winding’, and the medial part of the accessory piece appears to be markedly thinner than in *D. anoigeus* n. sp. Considering the different host (*M. terraesanctae*) and locality (Israel, Lake Galilee) recorded for *D. acanthobramae*, we do not consider these 2 species conspecific; however, *D. acanthobramae* requires redescription that should be based on new specimens collected from its type host and the type locality.

#### ***Dactylogyrus medicus*** **Řehulková n. sp.** (Fig. 4)

**Type- host:** *Garra rufa* (Heckel 1843) (Cyprinoidei: Cyprinidae).

**Figure 4.**
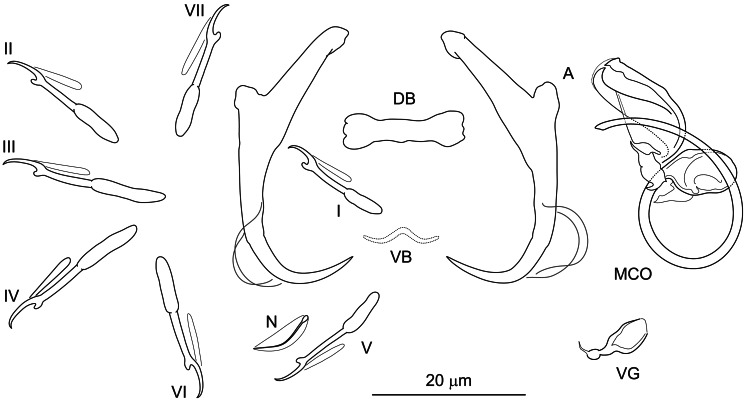
Hard structures of *Dactylogyrus medicus* n. sp. ex *Garra rufa*. A, anchor; DB, dorsal bar; VB, ventral bar; N, needle; I–VII, hooks; VG, vagina; MCO, male copulatory organ.

**Type-locality:** by the road Suleymania-Dukan, Little Zab, Sulaymaniyah Province, Iraq.

**Site on host:** Gill filaments.

**Type-material:** Holotype, 2 paratypes, 2 hologenophores (IPCAS M-791).

**Representative DNA sequence:** A nucleotide sequence of the partial gene for *28S* rRNA (729 bp long; OR817691), and nucleotide sequences representing a fragment (961 bp long; OR817710) including the partial gene for *18S* rRNA (467 bp), and the *ITS1* region (494 bp). No intraspecific variability was found.

**Infection indices:** Prevalence 60%, 1–6 monogeneans per infected host.

**Etymology:** The specific name refers to the fish host *G. rufa*, also known as the doctor fish.

**ZooBank registration (LSID):** urn:lsid:zoobank.org:act:6FAD82DA-C64D-4C48-A894-E6DC57FB340E.

**Description:** (Dimensions of the hard structures are given in Table 2.) Two pairs of anchors with elongate terminally tapering inner root, moderately developed outer root, proximally slightly swollen shaft and recurved point reaching level of tip of inner root. Dorsal bar straight, bone-shaped, with enlarged slightly indented ends. Ventral bar vestigial, poorly defined or absent, resembling an inverted flying bird symbol. One pair of needles located near hooks of pair V. Seven pairs of hooks, each with delicate point, protruded thumb and shank inflated along proximal half; FH loop extending to near level of shank inflation. MCO composed of basally articulated copulatory tube and accessory piece. Copulatory tube comprising bulbous base with flange and usually number 6-shaped shaft. Accessory piece articulated just posteriorly to base at level of the basal flange, closed leaf-shaped, serving as a guide for distal part of the tube. Vagina inconspicuous, lightly sclerotized, variable in shape.

**Differential diagnosis:** *Dactylogyrus medicus* n. sp. represents the fifth species of *Dactylogyrus* besides *D. tylognathi* Paperna, 1961, *D. garrae* Paperna, 1964 (Israel; Paperna, 1961, 1964), *D. acinacus* Gussev *et al*., 1993 and *D. rectotrabus* (Iran, Turkey; Gussev *et al*., 1993; Koyun, 2011) so far recorded on the doctor fish, *G. rufa.* A further 3 species of *Dactylogyrus* parasitizing species of *Garra*, i.e. *D. lingualis* Lang, 1981, *D. onychocirrus* Lang, 1981 and *D. spirotubivagina* Ann and Zang, 1988, have been described from *Garra orientalis* in China (Lang, 1981; Ann and Zhang, 1988). The haptoral configurations of all the *Dactylogyrus* species parasitizing *Garra* spp. show common features such as a rod-shaped dorsal bar, a missing or vestigial ventral bar, and anchors of the pseudanchoratus type (see Pugachev *et al*., 2009), which is characterized by a long inner root and short outer root, a swelling on the shaft and a point that is not well demarcated from the shaft (*vs* angularly demarcated from the shaft in the anchoratus type). *Dactylogyrus medicus* n. sp. is clearly differentiated from *D. garrae* and *D. tylognathi* parasitizing *G. rufa* and from all known *Dactylogyrus* spp. reported from *G. orientalis* (i.e. *D. lingualis*, *D. onychocirrus* and *D. spirotubivagina*) by having a shorter copulatory tube of the MCO (the copulatory tube is markedly longer and thinner, meandering or coiled in the 5 respective species). In this respect, *D. medicus* n. sp. is similar to *D. acinacus* and *D. rectotrabus*, which possess a relatively short J-shaped copulatory tube with a bulbous base. However, it clearly differs from the above 2 congeners by having an MCO with an accessory piece resembling a closed leaf (an accessory piece in the form of 1 or more rod-shaped plates placed in parallel to the copulatory tube). In addition, unlike in *D. acinacus* and *D. rectotrabus*, the ventral bar is present in *D. medicus* n. sp, although is barely visible, even under phase contrast optics.

*Dactylogyrus* spp. parasitizing *G. rufa* (i.e. *D. acinacus* and *D. medicus* n. sp.) are morphologically similar to *Dactylogyrus marocanus* (Fig. 2), a phylogenetically closely related parasite with a broad host range including torins and barbins in Morocco, as was previously suggested also by Řehulková *et al*. (2021).

#### ***Dactylogyrus regius*** **Řehulková n. sp.** (Fig. 5)

**Type-host:** *Chondrostoma regium* (Heckel 1843) (Leuciscidae: Leuciscinae).

**Figure 5.**
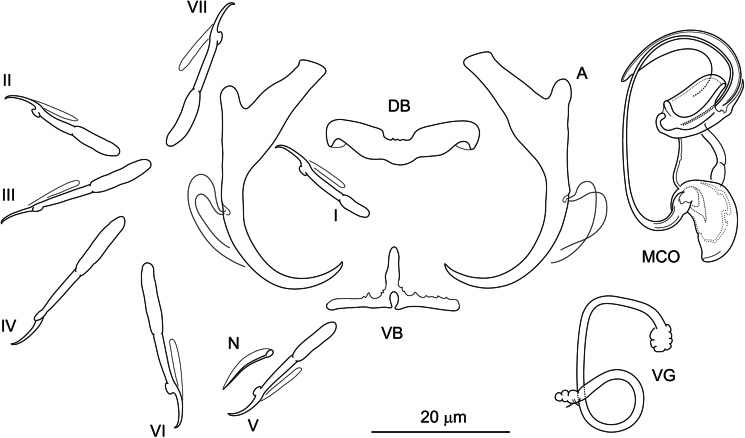
Hard structures of *Dactylogyrus regius* n. sp. ex *Chondrostoma regium*. A, anchor; DB, dorsal bar; VB, ventral bar; N, needle; I–VII, hooks; VG, vagina; MCO, male copulatory organ.

**Type-locality:** Du Choman, the Aw-e Shiler River, Sulaymaniyah Province, Iraq.

**Site on host:** Gill filaments.

**Type-material:** Holotype, 2 paratypes, 2 hologenophores (IPCAS M-792).

**Representative DNA sequence:** A nucleotide sequence of the partial gene for *28S* rRNA (703 bp long; OR817693), and nucleotide sequences representing a fragment (845 bp long; OR817707) including the partial gene for *18S* rRNA (467 bp), and the *ITS1* region (378 bp). No intraspecific variability was found.

**Infection indices:** Prevalence 83%, 1–8 monogeneans per infected host.

**Etymology:** The specific name refers to the fish host.

**ZooBank registration (LSID):** urn:lsid:zoobank.org:act:306AB571-A70D-4069-A964-DE265945723C.

**Description:** (Dimensions of hard structures are given in Table 2.) Two pairs of anchors with moderately long and terminally flattened inner root, rounded outer root and evenly curved shaft and point, point with slightly recurved tip and extending well past level of tip of inner root. Dorsal bar nearly yoke-shaped, with anteromedial depression. Ventral bar vestigial, inverted T-shaped, 3-armed. One pair of needles located near hooks of pair V. Seven pairs of hooks; each with delicate point, flattened thumb and shank inflated along proximal half; FH loop extending to near level of shank inflation. MCO composed of basally articulated copulatory tube and accessory piece. Copulatory tube with saclike base recurved posteriorly; shaft elongated, thin, nearly C-shaped. Accessory piece attached to base of tube as 2 filaments (1 markedly thinner) and formed distally as a plate-like sheath giving rise to tongue-shaped lobe directed backwards along the circle of the curved tube. Vagina a relatively long tube of variable course, with lobed ends.

**Differential diagnosis:** *Dactylogyrus regius* n. sp. belongs to the group of congeners having the MCO of the ʻchondrostomi' type, which is characterized by an accessory piece with a tongue-shaped lobe directed backwards along the circle of the curved copulatory tube. This morphological group includes parasites mostly of *Chondrostoma* hosts (e.g. *D. dirigerus*, *D. ergensi* and *D. globulatus*), *Telestes* hosts (e.g. *D. conchatus* and *D. sagittarius*) and *Alburnoides* hosts (e.g. *D. caucasicus* and *D. tissensis*) (Pugachev *et al*., 2009; Benovics *et al*., 2021*b*), which clustered together in the phylogenetic tree (Fig. 2). *Dactylogyrus regius* n. sp. differs from other congeners in the cluster by having the following combination of characters: sabre-shaped anchors, an inverted T-shaped ventral bar and an accessory piece of the MCO with a robust distal part appearing as plate-like sheath.

*Dactylogyrus regius* n. sp. most closely resembles *D. elegantis* (not included in our phylogenetic analyses), a parasite of *C. knerii*, *C. nasus* and *C. regium* (Stojanovski *et al*., 2004; Koyun, 2011; Benovics *et al*., 2018) in the comparative morphology of their haptoral structures. In both species, the anchors possess an evenly curved shaft and point (sabre-shaped type), straight to a broadly V-shaped dorsal bar with an anteromedial depression, and an inverted T-shaped ventral bar. *Dactylogyrus regius* n. sp. clearly differs from *D. elegantis* by having an accessory piece with a robust distal part appearing as a plate-like sheath through which the distal end of the copulatory tube passes (distal part simple, with groove in *D. elegantis*) and a proximal part formed as 2 filaments (proximal part appearing as a more compact bifurcated rod in *D. elegantis*).

#### ***Dactylogyrus rivalis*** **Řehulková n. sp.** (Fig. 6)

**Type-host:** *Squalius lepidus* Heckel 1843 (Cyprinoidei: Leuciscidae).

**Figure 6.**
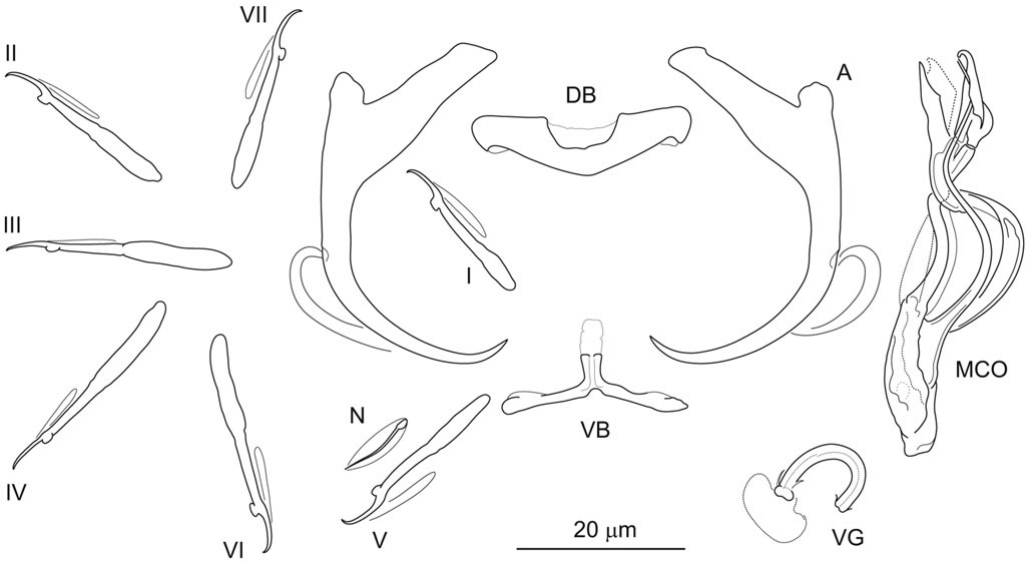
Hard structures of *Dactylogyrus rivalis* n. sp. ex *Squalius lepidus*. A, anchor; DB, dorsal bar; VB, ventral bar; N, needle; I–VII, hooks; VG, vagina; MCO, male copulatory organ.

**Type-locality:** Du Choman, Aw-e Shiler River, Sulaymaniyah Province, Iraq.

**Site on host:** Gill filaments.

**Type-material:** Holotype, 3 paratypes, 3 hologenophores (IPCAS M-793).

**Representative DNA sequence:** A nucleotide sequence of the partial gene for *28S* rRNA (730 bp long; OR817698), and nucleotide sequences representing a fragment (995 bp long; OR817715) including the partial gene for *18S* rRNA (487 bp), the *ITS1* region (488 bp) and *5.8S* region (20 bp). No intraspecific variability was found.

**Infection indices:** Prevalence 44%, 9–22 monogeneans per infected host.

**Etymology:** The specific name (an adjective) is from Latin (*rivalis* = a rival, competing) and refers to the co-occurrence of the new species with *D. vistulae* on the gills of *S. lepidus*.

**ZooBank registration (LSID):** urn:lsid:zoobank.org:act:206B6BE8-3767-419F-9662-48E1189F8D6B.

**Description:** (Dimensions of the hard structures are given in Table 2.) Two pairs of anchors with elongate and terminally flattened inner root, rounded outer root, markedly elongate shaft bent at its proximal third and short point not well demarcated from shaft and extending past level of tip of inner root. Dorsal bar nearly yoke-shaped, with anteromedial depression. Ventral bar vestigial, inverted T-shaped, 3-armed; anteromedial arm with lightly sclerotized termination; lateral arms resembling wings. One pair of needles located near hooks of pair V. Seven pairs of hooks; each with delicate point, flattened thumb and shank inconspicuously inflated along proximal half; FH loop extending to near level of shank inflation. MCO composed of basally articulated copulatory tube and accessory piece. Copulatory tube comprising elongate base and distally tapering sinusoidal shaft. Accessory piece bifurcated near midpoint into rod-shaped arm articulating to base of tube and membranous flap following convex curve of proximal half of the tube. Vagina a curled short tube.

**Differential diagnosis:** Until now only 3 species of *Dactylogyrus* have been recorded on *S. lepidus*, namely *D. dyki* Ergens and Lucký, 1959, *D. elegantis* and *D. vistulae* Prost, 1957 (Iraq; Abdullah and Abdullah, 2013). However, the microphotograph and drawings of the haptoral structures of *D. dyki* presented by the above authors show the anchors as having an elongate shaft markedly bent at its proximal third and poorly demarcated from the short point, which does not correspond to those originally described for *D. dyki* (anchors with moderately long shaft angularly demarcated from long point, i.e. the ʻwunderi' type in Pugachev *et al*., 2009). The configuration and morphology of the haptoral structures in *D. dyki* of Abdullah and Abdullah (2013), however, corresponds well to that in our specimens of *D. rivalis* n. sp. In addition, the measurements and overall morphology of the MCO and vagina reported by these authors match those of *D. rivalis* n. sp. Thus, considering that both parasites were found on the same host species and in close geographical proximity in Iraq, it is probable that the specimens identified by Abdullah and Abdullah (2013) as *D. dyki* were actually the new species described here as *D. rivalis* n. sp.

*Dactylogyrus rivalis* n. sp. shares a similar morphology of the dorsal and ventral bar with *Dactylogyrus* spp. occupying the same clade in the phylogenetic tree (see Fig. 2). Of these species, it most closely resembles *D. folkmanovae* in having anchors with a markedly elongate shaft bent at its proximal third and a short point not well demarcated from the shaft. It clearly differs from the latter species in possessing a sinusoidal copulatory tube (*vs* sickle-shaped copulatory tube in *D. folkmanovae*) supported by membranous accessory piece (*vs* more compact and distally bifurcated accessory piece in *D. folkmanovae*).

### Species diversity of *Gyrodactylus* parasites in Iraq

The diversity of the genus *Gyrodactylus* was found to be poorer when compared to *Dactylogyrus* diversity. *Gyrodactylus* spp. were collected from the gills, fins and skin of fish. Ten out of 13 cyprinoid species were parasitized by *Gyrodactylus* spp. A total of 12 *Gyrodactylus* species were identified and all of them were recognized as new to science, according to the autapomorphies in taxonomically important morphological characteristics and molecular phylogeny. The highest *Gyrodactylus* species diversity was recorded from *A. sellal*, collected from 3 localities; this host species was parasitized by 3 *Gyrodactylus* species at 3 localities (a maximum of 2 species were found from a single collection site, see below and Table 1). Two new *Gyrodactylus* species were collected from *G. rufa*, i.e. *G. satanicus* n. sp., and *G. vukicae* n. sp., with the former one exhibiting the highest prevalence across all collected *Gyrodactylus* species (*P* = 70%). The *G. iraqemembranatus* n. sp., which exhibited the widest host range among congeners, was collected from *A. sellal* (at 2 collection sites), and also from *Barbus lacerta* Heckel, 1843 and *P. trutta* (Heckel, 1843).

Due to there being insufficient material for morphological analyses of 4 species (i.e. a low number of mounted *Gyrodactylus* specimens or the presence of malformed taxonomically important characters), we properly describe here only 8 species below out of a total of 12. For 3 species, insufficient material was available for studying the morphology (i.e. *Gyrodactylus* sp. 2 from *L. barbulus*, *Gyrodactylus* sp. 3 from *A. sellal* and *Gyrodactylus* sp. 4 from *B. lacerta*); therefore, only orthologue sequences were deposited in GenBank, and were also included in the phylogenetic analyses. For the last species, *Gyrodactylus* sp. 1 collected from *P. trutta*, no DNA sequence data are available; therefore, it is only mentioned as ‘recorded’.

### Phylogenetic relationships of *Gyrodactylus* in Iraq

**Figure 7.**
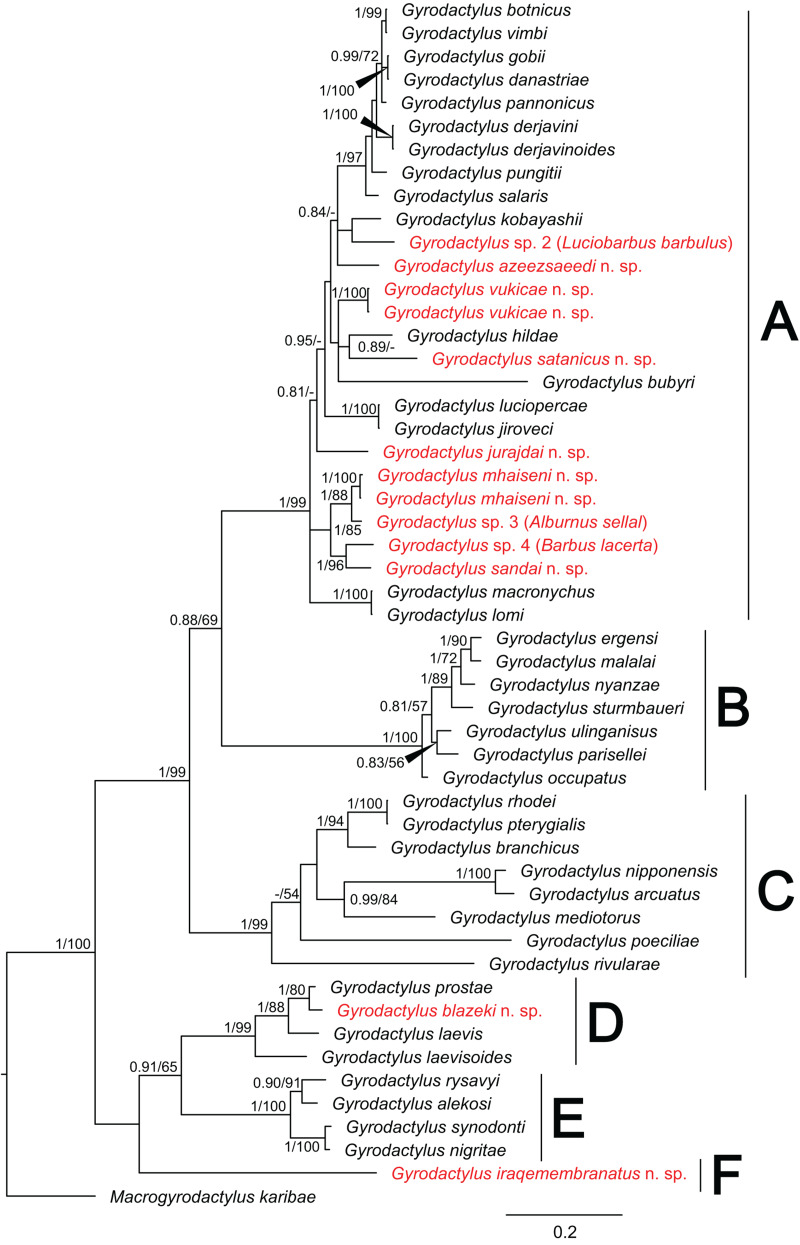
Phylogenetic tree of 49 *Gyrodactylus* spp. parasitizing various fish hosts. The tree is based on 52 combined sequences of partial *ITS1* and *ITS2* regions with *5.8S* rRNA, and rooted using *Macrogyrodactylus karibae*. Values at the nodes indicate posterior probabilities from BI and bootstrap values from ML analyses. Dashes indicate values below 0.70 and 50, respectively. Letters (A–F) represent specific well-supported clades or lineages. The newly described species from this study are in red.

The final concatenated nucleotide alignment comprising *ITS1*, *5.8S* and *ITS2* regions included 51 sequences of 49 *Gyrodactylus* species (for *G. mhaiseni* n. sp. and *G. vukicae* n. sp., 2 genetic variants were recorded and included in analyses) and spanned 858 unambiguously aligned nucleotide positions (285 bp for *ITS1*; 162 bp for *5.8S*; 411 bp for *ITS2*). Both phylogenetic analyses (BI and ML) generated trees with identical topologies and differed only partially in their nodal support (see the tree generated by BI in Fig. 7).

The phylogenetic analyses revealed that the investigated *Gyrodactylus* species belonged to 6 well-supported lineages. Lineage A encompassed 9 new *Gyrodactylus* species, congeners from Europe and also *G. kobayashii* Hukuda, 1940 parasitizing *C. auratus* from China and *G. hildae* García-Vásquez, Hansen, Christison, Bron and Shinn, 2011 parasitizing *Oreochromis niloticus* (Linnaeus, 1758) from Ethiopia. While the phylogenetic positions of *Gyrodactylus* sp. 1 from *L. barbulus*, *G. azeezsaeedi* n. sp., *G. vukicae* n. sp., *G. satanicus* n. sp. and *G. jurajdai* n. sp. within lineage A were not fully resolved, the other 4 newly described species formed a well-supported monophyletic group. Minor intraspecific variability was observed at the geographical level (for *G. mhaiseni* n. sp.) and the host species level (for *G. vukicae* n. sp.).

Lineage B included *Gyrodactylus* species parasitizing African freshwater fish of Cichlidae. The species belonging to lineage C were monogeneans of Palearctic (*G. arcuatus* Bychowsky, 1933, *G. branchicus* Malmberg, 1964, *G. nipponensis* Ogawa and Egusa, 1978, *G. rhodei* Žitňan, 1964 and *G. pterygialis* Bychowsky and Polyansky, 1953), Nearctic (*G. mediotorus* King, Marcogliese, Forest, McLaughlin and Bentzen, 2013) and Neotropic (*G. poeciliae* Harris and Cable, 2000) fish hosts. *Gyrodactylus blazeki* n. sp. grouped together with common Holarctic species within the lineage D. Lineage E encompassed 4 *Gyrodactylus* species parasitizing African silurids. *Gyrodactylus iraqemembranatus* n. sp., differing from other congeners by the morphology of taxonomically important characters (see above), has an unresolved relationship to the monophyletic group including lineages A–C and the monophyletic group including lineages D and E.

### Morphological and molecular characterization of the new *Gyrodactylus* species

#### ***Gyrodactylus azeezsaeedi*** **Rahmouni n. sp.** (Fig. 8)

**Type-host:** *Squalius berak* Heckel, 1843 (Cyprinoidei: Leuciscidae)

**Figure 8.**
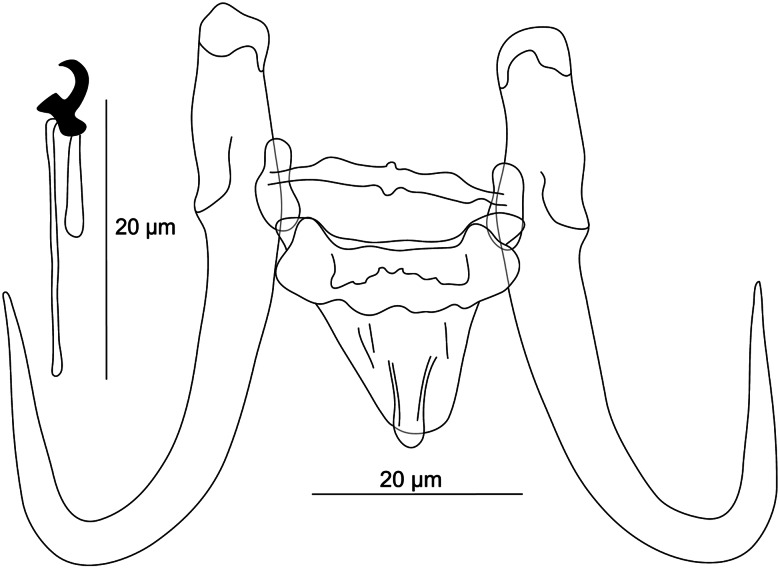
Hard structures of haptor of *Gyrodactylus azeezsaeedi* n. sp. ex *Squalius berak*.

**Type-locality:** Kani Shok, a tributary of the Tabin River, Sulaymaniyah Province, Iraq

**Site on host:** Fins

**Type material:** Holotype and 2 paratypes (IPCAS M-782).

**Representative DNA sequence**: A nucleotide sequence representing a fragment (1274 bp long; OR773093) including the partial *ITS1* region (684 bp long), *5.8S* rDNA (157 bp) and the partial *ITS2* region (433 bp). No intraspecific variability was found.

**Infection indices:** Prevalence 10%, 1–5 monogeneans per infected host.

**Etymology:** The specific name ‘*azeezsaeedi*’ honours Dr Mohammed Azeez Saeed, the coordinator of international cooperation at Salahaddin University (Erbil, Iraq), for his precious help with the organization of the field trip and hospitality.

**ZooBank registration (LSID):** urn:lsid:zoobank.org:act:5139D2BB-0B0A-45F6-8D80-76F88C41D9E4.

**Description:** (Dimensions of the hard structures are given in Table 3.) Haptor subcircular; tips of inner roots with hat-like cover; base with posterior folds; root relatively long and straight; shaft slightly bowed; point curved and elongated. Ventral bar with blunt and short bilateral processes extending out of bar; median part may show a hollow; membrane slightly trapezoid, almost 2/3 length of hamuli shaft, with striations ending posteriorly in a median ridge. Dorsal bar simple, with projections at halfway point and attenuated ends inserted into terminal plates. Marginal hooks with a flat base, globose heel slightly curved outward, conspicuous finger-like toe inward, conspicuous shelf, curved point, and sickle proper approximately perpendicular to terminal edge of toe, gently curved downward to a point slightly exceeding the toe; filament loop extending about 1/2 handle length. MCO not observed.

**Table 3.** Morphometric data for newly described *Gyrodactylus* species

*Gyrodactylus* species	*G. azeezsaeedi* n. sp.	*G. blazeki* n. sp.	*G. iraqemembranatus* n. sp.	*G. jurajdai* n. sp.	*G. mhaiseni* n. sp.	*G. sandai* n. sp.	*G. satanicus* n. sp.	*G. vukicae* n. sp.
Host species	*S. berak*	*Alburnus* sp.	*P. trutta*	*C. regium*	*A. sellal*	*C. umbla*	*G. rufa*	*G. rufa*
			*A. sellal*			*C. macrostomum*		
			*B. lacerta*					
Anchors								
Total length	55.7 (54.8–57.3)_8_	31.9 (31.6–32.1)_3_	24 (23–25)_16_	63.9 (57.2–74.5)_5_	56.5 (51.7–59.9)_10_	102.7 (100–105.1)_10_	62.6 (61.5–63.5)_10_	44.9 (43.9–46.1)_4_
Root	16.5 (15.5–17.1)_8_	10.6 (9.9–11.3)_3_	8 (6–10)_16_	19.5 (17.8–22.2)_5_	17.4 (14.1–19.4)_10_	33 (30.1–34.2)_10_	21.5 (20.1–22.8)_10_	14.6 (13.8–15.1)_4_
Shaft	43.1 (42–44.1)_8_	26.2 (25.8–26.4)_3_	19 (18–21)_16_	48.2 (42.8–54.6)_5_	42.6 (39.9–44.8)_10_	74.6 (73.4–75.5)_10_	44.2 (43.9–45)_10_	32.9 (32–33.5)_4_
Point	27.3 (26.3–27.9)_8_	13.6 (13.4–13.8)_3_	9 (7.5–11)_16_	29.4 (24.4–36)_5_	25.3 (22.4–27.3)_10_	43.3 (42.1–45)_10_	28.8 (27.2–29.8)_10_	22.4 (21.8–22.8_4_)
Transverse bar								
Total length	24.9 (24–26.1)_8_	14.6 (14.1–15)_3_	3 (2–8)_16_	26.9 (22–33.4)_5_	23 (19.8–25.6)_10_	62.1 (58.5–63.9)_10_	31.7 (30.8–32.8)_10_	18.7 (17.6–19.7)_4_
Total width	24.5 (23.–25.3)_8_	11.9 (11.6–12.1)_3_	7 (2–9)_16_	22.5 (21.3–23.7)_5_	23.3 (10.2–25.4)_10_	57.6 (53.9–59.8)_10_	23.6 (22.8–24.5)_10_	16.9 (16.6–17.3)_4_
Tips length	2.9 (2.2.–3.6)_8_	0	0	2 (1.7–2.8)_5_	2.1 (1.6–2.5)_10_	19.6 (16.6–22.6)_10_	3 (2.7–3.3)_10_	1.6 (1.4–2)_4_
Median width	6.2 (5.6–7)_8_	4.2 (4–4.6)_3_	0	7.2 (6.4–8.4)_5_	5.5 (4.1–6.5)_10_	11.7 (10.5–12.6)_10_	6 (5.7–6.2)_10_	4.5 (3.8–5.2)_4_
Membrane length	15 (14–15.9)_8_	8.5 (8.3–8.6)_3_	0	18.8 (12.5–29.5)_5_	14.5 (12.8–15.7)_10_	30.1 (28.6–31.3)_10_	22.3 (21.8–23)_10_	11.5 (11.3–11.8)_4_
Membrane width	14.5 (14–15.2)_8_	3.7 (3.6–3.8)_3_	0	15.1 (12.2–20.3)_5_	13.8 (10.6–16.6)_10_	22.7 (21.3–25.2)_10_	16.8 (16.1–17.3)_10_	9.5 (8.4–10.5)_4_
Dorsal bar								
Total length	26.5 (25.7–27.3)_8_	10.9 (10–11.6)_3_	7 (6.8–8.5)_16_	26.8 (24.1–31.3)_5_	23.5 (19.9–25)_10_	49 (47–55)_10_	27.2 (26.7–27.6)_10_	18.6 (17.8–19.7)_4_
Width at midpoint	2 (1.7–2.6)_8_	1.4 (1.4–1.5)_3_	1 (0.7–1.2)_16_	1.8 (1.5–2.3)_5_	1.8 (1.1–2.4)_10_	2.4 (1.9–3.5)_10_	2.3 (2.1–2.4)_10_	1.5 (1.4–1.6)_4_
Marginal hooks								
Total length	24.2 (23.5–25.2)_8_	20.3 (19.9–20.5)_3_	11.8 (11–12.9)_16_	24 (22.8–27.3)_5_	25.7 (23.2–27.4)_10_	45.9 (45–46.5)_10_	27.4 (26.8–28)_10_	23 (22.5–23.4)_4_
Filament loop length	9.1 (8.1–10.1)_8_	10.5 (9.6–11.2)_3_	6 (4.2–8)_16_	10.5 (9.6–11.2)_5_	8.5 (6.9–9.3)_10_	12.2 (10.2–14.6)_10_	8 (7.5–8.8)_10_	7.5 (6.8–8.2)_4_
Handle length	18.4 (17.5–19)_8_	14.8 (14.5–15.1)_3_	8.7 (6.9–9.5)_16_	20.2 (18.3–23.3)_5_	20.1 (17.7–21.6)_10_	38.2 (37.4–39–1)_10_	21.2 (21.1–21.5)_10_	17.6 (17.3–18.1)_4_
Sickle length to shaft attachment	4.8 (4.1–5.9)_8_	6.3 (6.1–6.4)_3_	2.9 (2.1–3.5)_16_	5.4 (4.9–6)_5_	5.6 (4.9–6.2)_10_	8.4 (5.5–9.7)_10_	6.6 (6.2–6.9)_10_	5.6 (5.4–5.7)_4_
Sickle proximal width	3.6 (3.1–4)_8_	2.9 (2.3–3.5)_3_	2 (1.7–2.8)_16_	3.5 (3.1–4.1)_5_	3.5 (2.8–4.1)_10_	6.5 (5.9–7.1)_10_	3.7 (3.5–3.8)_10_	3.1 (2.8–3.5)_4_
Sickle distal width	4.4 (4–4.9)_8_	4.5 (4.2–4.6)_3_	2.1 (1.8–2.8)_16_	3.9 (3.4–4.8)_5_	3.9 (2.8–4.5)_10_	6.9 (6.4–7.8)_10_	5.2 (5.1–5.5)_10_	3.8 (3.6–3.9)_4_
Shaft length of sickle	3.8 (3–4.4)_8_	3.7 (3.6–3.8)_3_	1.8 (1.5–2.2)_16_	4.1 (3.6–4.6)_5_	3.8 (3.2–4.3)_10_	6.8 (6.1–7.6)_10_	5 (4.7–5.2)_10_	3.3 (3.3–3.5)_4_
Point length of sickle	1.7 (1.4–2)_8_	1.4 (1.3–1.5)_3_	0.9 (0.7–1.2)_16_	1.3 (1.1–1.7)_5_	1.6 (1.4–1.8)_10_	3.2 (2.6–3.9)_10_	1.2 (1.1–1.4)_10_	1.4 (1.3–1.5)_4_

The first number represents the mean value and is followed by the range of obtained measurements in brackets. The lower index number represents the number of measured specimens.

**Differential diagnosis:** Herein, *Gyrodactylus* from *S. berak* was identified for the first time. The haptoral morphology exhibited by *G. azeezsaeedi* n. sp. resembles that of *G. gobii* (Schulman, 1953) parasitizing widespread *Gobio gobio* (Linnaeus, 1758); *G. leucisci* Žitňan, 1964 and *G. osoblahensis* Ergens, 1963, mostly parasitizing *Leuciscus leuciscus* (Linnaeus, 1758) and *S. cephalus*; and, finally, *G. scardiniensis* Glaser, 1974 from *Scardinius erythrophthalmus* and *Scardinius cephalus* (Ergens, 1991, 1992; Pugachev *et al*., 2009). The new species differs from *G. gobii* by the shape and size of its dorsal bar, which shows projections at the halfway point, a feature missing in *G. gobii*, and which is comparatively longer in *G. azeezsaeedi* n. sp. *Gyrodactylus azeezsaeedi* n. sp. is distinguishable from *G. leucisci* in having (i) shorter hamuli (54.8–57.3 *μ*m in *G. azeezsaeedi* n. sp. *vs* 63.0–73.0 *μ*m in *G. leucisci*), (ii) a longer dorsal bar (25.7–27.3 *μ*m in *G. azeezsaeedi* n. sp. *vs* 19.0–21.0 *μ*m in *G. leucisci*) and (iii) shorter marginal hooks (23.5–25.2 *μ*m in *G. azeezsaeedi* n. sp. *vs* 30.0–31.0 *μ*m in *G. leucisci*). *Gyrodactylus azeezsaeedi* differs from *G. osoblahensis* in having (i) shorter hamuli (54.8–57.3 *μ*m in *G. azeezsaeedi* n. sp. *vs* 60.0–70.0 *μ*m in *G. osoblahensis*), (ii) a shorter ventral bar (24.0–26.1 *μ*m in *G. azeezsaeedi* n. sp. *vs* 29.0–33.0 *μ*m in *G. osoblahensis*) associated to (iii) a longer membrane (14.0–15.9 *μ*m in *G. azeezsaeedi* n. sp. *vs* 22–26 *μ*m in *G. osoblahensis*) and finally (iv) shorter marginal hooks (23.5–25.2 *μ*m in *G. azeezsaeedi* n. sp. *vs* 31.0–39.0 *μ*m in *G. osoblahensis*). It is further discriminated from *G. scardiniensis* by its (i) shorter hamuli (54.8–57.3 *μ*m in *G. azeezsaeedi* n. sp. *vs* 60.0–70.0 *μ*m in *G. scardiniensis*), and (ii) shorter marginal hooks (23.5–25.2 *μ*m in *G. azeezsaeedi* n. sp. *vs* 32.0–38.0 *μ*m in *G. scardiniensis*).

#### ***Gyrodactylus blazeki*** **Rahmouni n. sp.** (Fig. 9)

**Type-host:** *Alburnus* sp. (Cyprinoidei: Cyprinidae)

**Figure 9.**
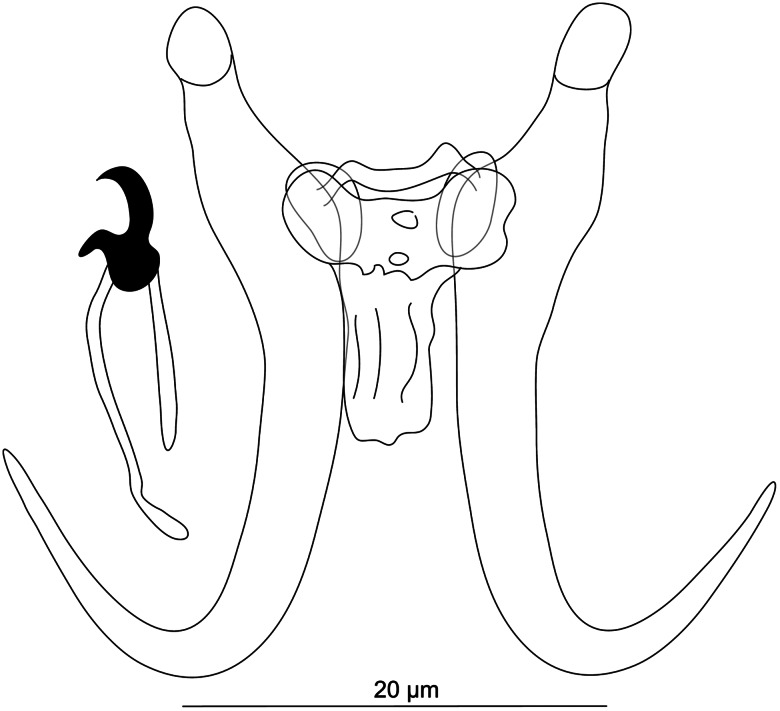
Hard structures of haptor of *Gyrodactylus blazeki* n. sp. ex *Alburnus* sp.

**Type-locality:** Grdi Go, Zalm stream, Sulaymaniyah Province, Iraq

**Site on host:** Gill filaments

**Type material:** Holotype and 1 paratype (IPCAS M-783).

**Representative DNA sequence:** A nucleotide sequence representing a fragment (895 bp long; OR773085) including the partial *ITS1* region (347 bp long), *5.8S* rDNA (157 bp) and the partial *ITS2* region (391 bp). No intraspecific variability was found.

**Infection indices:** Prevalence 5%, 3 monogeneans per infected host.

**Etymology:** The specific name ‘*blazeki*’ honours the ichthyologist Dr Radim Blažek from the Institute of Vertebrate Biology, Czech Academy of Sciences (Brno, Czech Republic) in recognition of his past research on *Gyrodactylus*.

**ZooBank registration (LSID):** urn:lsid:zoobank.org:act:2F14F7A0-277D-4DA3-9224-9AC9EAE84E42.

**Description:** (Dimensions of the hard structures are given in Table 3.) Haptor subcircular; tips of inner roots with hat-like cover; base with no posterior folds; root relatively long; shaft slightly bowed; point curved and elongated. Ventral bar lacking bilateral processes; median part of a common width may show holes; membrane slightly rectangular, almost 1/3 length of hamuli shaft, with striations. Dorsal bar simple, with posteriorly directed projections and attenuated ends inserted into terminal plates. Marginal hooks with prominent globose heel, curved finger-like toe downward, conspicuous shelf, curved point, sickle proper curved downward to a point slightly exceeding the toe; filament loop extending about handle length. MCO not observed.

**Differential diagnosis:** This study presents the first data on monogeneans parasitizing *Alburnus* spp. from the Middle East. So far, no *Gyrodactylus* species with similar haptoral morphology has been reported in the Middle East. The overall morphology exhibited by *G. blazeki* n. sp. resembles that of *G. laevis* Malmberg, 1957 and *G. prostae* Ergens, 1963, both known from a range of Palearctic cyprinids (Pugachev *et al*., 2009). This resemblance is seen in the shape of the hamuli with well-developed roots with folds, the ventral bar lacking bilateral processes and marginal hooks with a well-developed heel. Compared to *G. laevis, G. blazeki* n. sp. possesses a shorter ventral bar membrane (8.3–8.6 *μ*m in *G. blazeki* n. sp. *vs* 9–16 *μ*m in *G. laevis*). *Gyrodactylus blazeki* n. sp. differs from *G. prostae* in having (i) shorter hamuli (31.6–32.1 *μ*m in *G. blazeki* n. sp. *vs* 44.0–60.0 *μ*m in *G. prostae*), (ii) shorter ventral bar membrane (8.3–8.6 *μ*m in *G. blazeki* n. sp. *vs* 12.0–16.0 *μ*m in *G. prostae*) and (iii) shorter marginal hooks (19.9–20.5 *μ*m in *G. blazeki* n. sp. *vs* 24.0–30.0 *μ*m in *G. prostae*).

#### ***Gyrodactylus iraqemembranatus*** **Rahmouni n. sp.** (Fig. 10)

**Type-host:** *Paracapoeta trutta* (Heckel, 1843) (Cyprinoidei: Cyprinidae)

**Figure 10.**
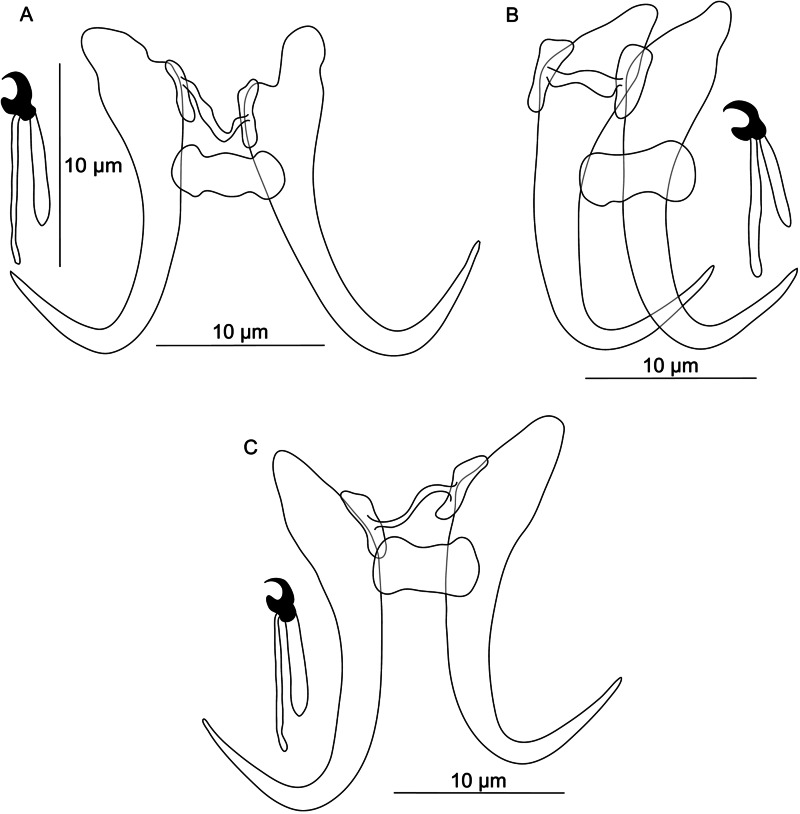
Hard structures of haptor of *Gyrodactylus iraqemembranatus* n. sp. ex *Paracapoeta trutta* (A), ex *Alburnus sellal* (B), ex *Barbus lacerta* (C).

**Additional hosts:** *Alburnus sellal* Heckel, 1843 (Cyprinoidei: Leuciscidae), *Barbus lacerta* Heckel, 1843 (Cyprinoidei: Cyprinidae)

**Type-locality:** Kani Shok, tributary of Tabin River, Sulaymaniyah Province, Iraq

**Additional locality:** wadi Kalat Shirah, a tributary of the Tabin River, and the Tabin River in Zahrzi for *A. sellal*; Kani Shok, a tributary of the Tabin River also for *B. lacerta*, all localities in Sulaymaniyah, Iraq

**Site on host:** Gill filaments for *P. trutta* and *B. lacerta*, fins for *A. sellal*.

**Type material:** Holotype and 6 paratypes (IPCAS M-784/1-3).

**Representative DNA sequence:** A nucleotide sequence representing a fragment (905 bp long; OR773087) including the partial *ITS1* region (370 bp long), *5.8S* rDNA (157 bp) and the partial *ITS2* region (378 bp). No intraspecific variability was found.

**Infection indices:** prevalence at type host 30%, 8–41 monogeneans per infected host.

**Etymology:** The specific name ‘*iraqemembranatus*’ refers to the morphological similarity between the new species found on cyprinid hosts from Iraq and its previously described congener *G. emembranatus* Malmberg, 1970, whose specific name refers to the absence of the membrane in the ventral bar.

**ZooBank registration (LSID):** urn:lsid:zoobank.org:act:B4738C07-9748-4217-80C0-D5510AC31E4F.

**Description:** (Dimensions of the hard structures are given in Table 3.) Haptor subcircular; tips of inner root uncovered; base with no posterior folds; root short; shaft slightly bowed; point curved and elongated. Ventral bar lacking bilateral processes and membrane; median part of a common width. Dorsal bar constricted at halfway point, with attenuated ends inserted into terminal plates. Marginal hooks with a flat base, circular heel downward, blunt toe slightly outward, no shelf, curved point, sickle proper approximately perpendicular to the base, gently curved downward to a point approximately the same level of toe; filament loop extending almost handle length. MCO with single prominent apical spine and row of at least 10 spinelets.

**Differential diagnosis:** Previous parasitological investigations performed by Al-Sa'adi (2007) on *A. sellal*, a native leuciscid inhabiting watersheds in Iraq, revealed the presence of specimens that were assigned to *G. sprostonae* Ling, 1962 (Mhaisen and Abdul-Ameer, 2013). The original work of Al-Sa'adi (2007) was unavailable for us to check the validity of this assignment. Considering the overall morphology of *G. sprostonae*, known so far from a range of widespread cyprinids (Pugachev *et al*., 2009), the newly described *G. iraqemembranatus* n. sp. from *A. sellal* differs considerably from the former species in having (i) shorter hamuli (23.1–25.0 *μ*m in *G. iraqemembranatus* n. sp. *vs* 41.0–62.0 *μ*m in *G. sprostonae*) and (ii) a shorter ventral bar (2.7–8.2 *μ*m in *G. iraqemembranatus* n. sp. *vs* 13.0–26.0 *μ*m in *G. sprostonae*) with no membrane (*vs* well-developed membrane in *G. sprostonae*). The distinction between *G. iraqemembranatus* n. sp. and *G. sprostonae* was further supported by genetic data (Fig. 7). Likewise, specimens identified as *G. sprostonae* were also reported on *B. lacerta* occurring in the Tajan River, together with *G. ctenopharyngodonis* Ling, 1962 (both localities in Iran), but no drawings of the haptoral structures were included (Barzegar *et al*., 2018). With regard to meristic data available in Barzegar *et al*. (2018), *G. iraqemembranatus* n. sp. is highly distinguishable from *G. ctenopharyngodonis* in having (i) shorter hamuli (23.1–25.0 *μ*m in *G. iraqemembranatus* n. sp. *vs* 53.8–54.5 *μ*m in *G. ctenopharyngodonis*), (ii) a shorter ventral bar (2.7–8.2 *μ*m in *G. iraqemembranatus* n. sp. *vs* 20.7–21.2 *μ*m in *G. ctenopharyngodonis*) with no membrane, (iii) a shorter dorsal bar (6.8–8.5 *μ*m in *G. iraqemembranatus* n. sp. *vs* 15.4–16.1 *μ*m in *G. ctenopharyngodonis*) and (iv) shorter marginal hooks (11.0–12.9 *μ*m in *G. iraqemembranatus* n. sp. *vs* 24.6–25.2 *μ*m in *G. ctenopharyngodonis*). Hitherto, *G. elegans* (Nordmann, 1832) was reported by Nasraddin (2013) on *P. trutta* inhabiting the Middle East (Iraq) (Mhaisen and Abdul-Ameer, 2013), and eastern Anatolia (Turkey) (Koyun *et al*., 2019). Since no morphology of the haptoral apparatus of *G. elegans* from *P. trutta* has so far been detailed, it remains hard to know whether the previously collected specimens truly corresponded to *G. elegans*. This latter species has been repeatedly misidentified and many researchers have randomly assigned dozens of species to *G. elegans* (see remarks in Malmberg, 1970; Pugachev *et al*., 2009). *Gyrodactylus iraqemembranatus* n. sp. resembles *G. elegans* regarding the ventral bar, which lacks bilateral processes. Besides the size of the haptoral sclerotized structures, the main differences between *G. iraqemembranatus* n. sp. and *G. elegans* are in (i) the shape of the hamuli, which have poorly developed roots in *G. iraqemembranatus* n. sp. (*vs* well-developed roots in *G. elegans*) and in (ii) the ventral bar membrane, which is absent in *G. iraqemembranatus* n. sp. (vs the presence of a spine-like shaped membrane in *G. elegans*) (Malmberg, 1970). The distinction between *G. iraqemembranatus* n. sp. and *G. elegans* was further supported by genetic data (Fig. 7). *Gyrodactylus markevitschi* Kulakovskaya, 1951 was first described from European *Barbus barbus* (Linnaeus, 1758), then later reported from distinct west Asian locations (Iraq) (Mhaisen and Abdul-Ameer, 2013; Koyun *et al*., 2019). It was also recorded on a range of Palearctic cyprinids (Pugachev *et al*., 2009). On the basis of merisitic data available in Pugachev *et al*. (2009), *G. iraqemembranatus* n. sp. differs from *G. markevitschi* in having (i) shorter hamuli (23.1–25.0 *μ*m in *G. iraqemembranatus* n. sp. *vs* 56–58 *μ*m in *G. markevitschi*), (ii) a shorter ventral bar (2.7–8.2 *μ*m in *G. iraqemembranatus* n. sp. *vs* 22.0–25.0 *μ*m in *G. markevitschi*) with no bilateral processes, (iii) a shorter dorsal bar (6.8–8.5 *μ*m in *G. iraqemembranatus* n. sp. *vs* 18.0–20.0 *μ*m in *G. markevitschi*) and finally (iv) shorter marginal hooks (11.0–12.9 *μ*m in *G. iraqemembranatus* n. sp. *vs* 26.0–29.0 *μ*m in *G. markevitschi*).

In this study, *G. iraqemembranatus* n. sp. showed intraspecific variability in the size of the ventral bar, where the longest and narrowest ventral bar was observed in specimens parasitizing *A. sellal* (see Table 3).

#### ***Gyrodactylus jurajdai*** **Rahmouni n. sp.** (Fig. 11)

**Type-host:** *Chondrostoma regium* (Heckel, 1843) (Cyprinoidei: Leuciscidae)

**Figure 11.**
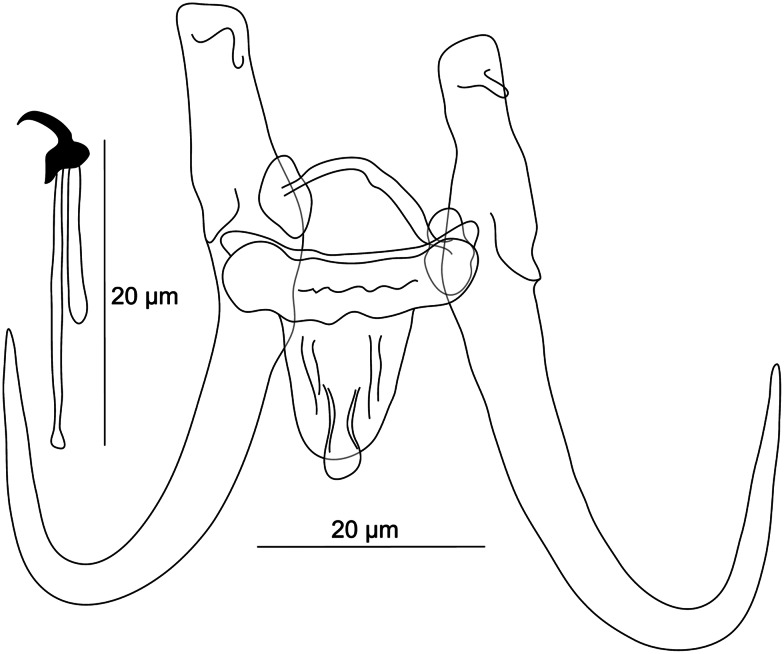
Hard structures of haptor of *Gyrodactylus jurajdai* n. sp. ex *Chondrostoma regium*.

**Type-locality:** Du Choman, the Aw-e Shiller River, Sulaymaniyah Province, Iraq

**Site on host:** Fins

**Type material:** Holotype and 1 paratype (IPCAS M-785).

**Representative DNA sequence:** A nucleotide sequence representing a fragment (1117 bp long; OR773088) including the partial *ITS1* region (546 bp long), *5.8S* rDNA (157 bp) and the partial *ITS2* region (414 bp). No intraspecific variability was found.

**Infection indices:** Prevalence 50%, 1–2 monogeneans per infected host.

**Etymology:** The specific name ‘*jurajdai*’ honours the ichthyologist Dr Pavel Jurajda from the Institute of Vertebrate Biology, Czech Academy of Sciences (Brno, Czech Republic) in recognition of his crucial contribution to parasitological work.

**ZooBank registration (LSID):** urn:lsid:zoobank.org:act:CA4D2FD9-E78F-4BCF-BE22-5CAC37EE457D.

**Description:** (Dimensions of the hard structures are given in Table 3.) Haptor subcircular; tips of inner root with narrow wart-like projections anteriorly; base with posterior folds; relatively long; shaft slightly bowed; point curved and elongated. Ventral bar with blunt, short, triangular bilateral processes extending out of bar; median part may show a hollow; membrane oval, almost 2/3 length of hamuli shaft, with striations ending posteriorly in a median ridge. Dorsal bar curved, slightly swollen at halfway point, with attenuated ends inserted into terminal plates. Marginal hooks with flat globose heel, elongate toe and curved downward, conspicuous shelf, curved point, sickle proper gently curved downward to a point approximately perpendicular to toe shelf; filament loop extending almost 2/3 handle length. MCO not observed.

**Differential diagnosis:** We present herein the first morphological characterization of a gyrodactylid species from *C. regium*. *Gyrodactylus jurajdai* n. sp. resembles its Eurasian relatives known from *Chondrostoma* spp. regarding the haptoral sclerotized structures – specifically, *G. chondrostomi* Ergens, 1967 and *G. macrocornis* Ergens, 1963, both parasitizing *C. nasus* (Linnaeus, 1758), and *G. derjavini* Mikailov, 1975 from *Chondrostoma oxyrhynchum* Kessler, 1877 (Pugachev *et al*., 2009). *Gyrodactylus jurajdai* n. sp. differs from *G. chondrostomi* in having (i) longer hamuli (57.2–74.5 *μ*m in *G. jurajdai* n. sp. *vs* 38.0–40.0 *μ*m in *G. chondrostomi*), (ii) shorter ventral bar (22.0–33.4 *μ*m in *G. jurajdai* n. sp. *vs* 18.0–19.0 *μ*m in *G. chondrostomi*) and (iii) longer dorsal bar (24.1–31.3 *μ*m in *G. jurajdai* n. sp. *vs* 15.0–17.0 *μ*m in *G. chondrostomi*), and (iv) differently shaped sickle of marginal hooks with a conspicuous shelf in *G. jurajdai n.* sp. in comparison to that in *G. chondrostomi*. The new species is discriminated from *G. macrocornis* by having (i) a ventral bar possessing a ridge (ridge missing in *G. macrocornis*) and (ii) relatively shorter hamuli (57.2–74.5 *μ*m in *G. jurajdau* n. sp. *vs* 74.0–58.0 *μ*m in *G. macrocornis*).

#### ***Gyrodactylus mhaiseni*** **Rahmouni n. sp.** (Fig. 12)

**Type-host:** *Alburnus sellal* Heckel, 1843 (Cyprnoidei: Leuciscidae)

**Figure 12.**
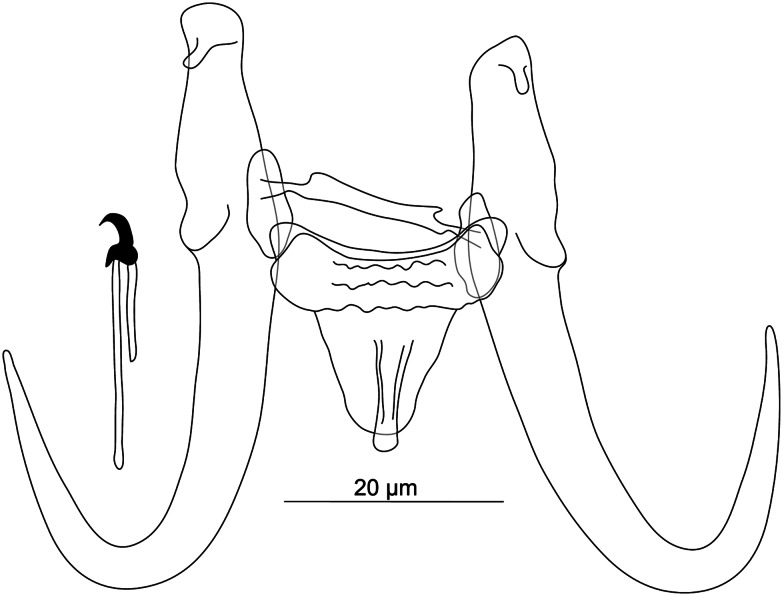
Hard structures of haptor of *Gyrodactylus mhaiseni* n. sp. ex *Alburnus sellal*.

**Type-locality:** wadi Kalat Shirah, a tributary of the Tabin River, Sulaymaniyah Province, Iraq

**Additional locality:** Zahrzi in Tabin River, Sulaymaniyah Province, Iraq

**Type material:** Holotype and 2 paratypes (IPCAS M-786).

**Site on host:** Fins

**Representative DNA sequence:** A nucleotide sequence representing a fragment (1148 bp long; OR773082) including the partial *ITS1* region (551 bp long), *5.8S* rDNA (157 bp) and the partial *ITS2* region (440 bp). Minor intraspecific variability was found between 2 host populations (*ITS1*, *p-*distance = 0.4%; *ITS2*, *p-*distance = 0.3%).

**Infection indices:** Prevalence at the type locality 25%, 1–4 monogeneans per infected host.

**Etymology:** The specific name ‘*mhaiseni*’ honours the parasitologist Professor Furhan T. Mhaisen in recognition of his crucial contribution to parasitological work on marine and freshwater fishes in the Middle East.

**ZooBank registration (LSID):** urn:lsid:zoobank.org:act:31283AC6-1CD4-450B-998F-A1D5042C97E5.

**Description:** (Dimensions of the hard structures are given in Table 3.) Haptor subcircular; tips of inner root with narrow wart-like projections anteriorly; base with posterior folds; root long; shaft slightly bowed; point curved and elongated. Ventral bar with blunt, short, almost triangular bilateral processes extending out of bar; median part with a hollow; membrane slightly trapezoid, almost 1/2 length of hamuli shaft, with striations ending posteriorly in a median ridge. Dorsal bar straight, with projections near extremities and attenuated ends inserted into terminal plates. Marginal hooks with globose downward heel, elongate toe and curved downward, conspicuous shelf, curved point, sickle proper approximately perpendicular to base, gently curved downward to a point slightly perpendicular to toe shelf; filament loop extending almost 1/2 handle length. MCO not observed.

**Differential diagnosis:** In addition to *G. iraqemembranatus* n. sp., *A. sellal* hosted another species recognized as new to science, namely *G. mhaiseni* n. sp. These 2 species are easily distinguishable regarding the morphotype of the hamuli, comprising long roots in *G. mhaiseni* n. sp. unlike the poorly developed ones in *G. iraqemembranatus* n. sp., as well as that of the ventral bar, showing bilateral processes and a long membrane with a ridge in the former species, but the absence of these features in *G. iraqemembranatus* n. sp. According to genetic data, slight intraspecific variability in haptoral sclerites was observed at the geographical scale. With respect to *G. sprostonae*, a species already reported from *A. sellal* (Iraq) (Mhaisen and Abdul-Ameer, 2013), measurements of the haptoral sclerites overlap those of *G. mhaiseni* n. sp. The main differences between these 2 species are (i) the projections on the dorsal bar, (ii) the median ridge in the ventral bar membrane and (iii) the thick shaft of the hook sickle in *G. mhaiseni* n. sp., all features missing in *G. sprostonae* (Pugachev *et al*., 2009).

#### ***Gyrodactylus sandai*** **Rahmouni n. sp.** (Fig. 13)

**Type-host:** *Capoeta umbla* (Heckel, 1843) (Cyprinoidei: Cyprinidae)

**Figure 13.**
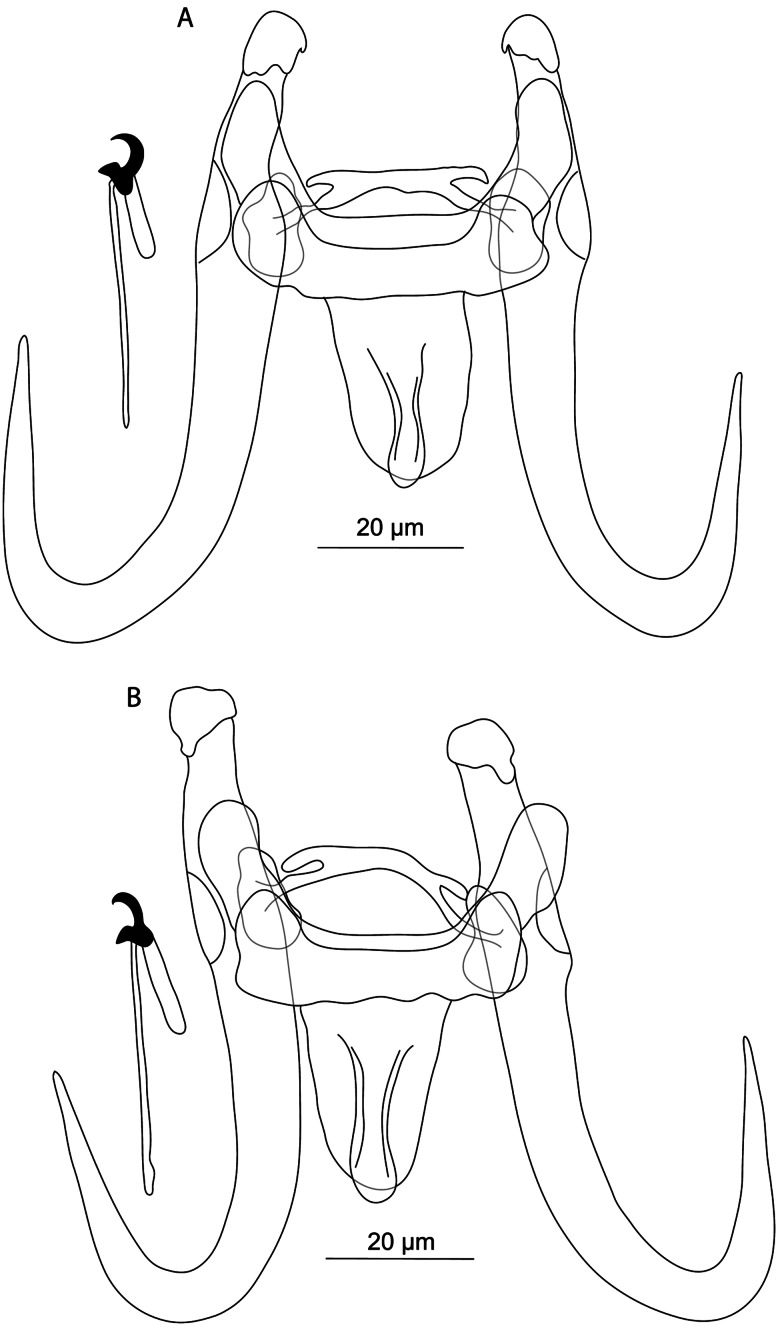
Hard structures of haptor of *Gyrodactylus sandai* n. sp. ex *Capoeta umbla* (A), ex *Cyprinion macrostomum* (B).

**Type-locality:** wadi Kalat Shirah, tributary of the Tabin River, Sulaymaniyah Province, Iraq

**Additional hosts:** *Cyprinion macrostomum* Heckel, 1843 (Cyprinoidei: Cyprinidae)

**Site on host:** Fins

**Type material:** Holotype and 2 paratypes (IPCAS M-787/1-2).

**Representative DNA sequence:** A nucleotide sequence representing a fragment (1120 bp long; OR773089) including the partial *ITS1* region (491 bp long), *5.8S* rDNA (157 bp) and the partial *ITS2* region (472 bp). No intraspecific variability was found.

**Infection indices:** Prevalence at type host 50%, 1–2 monogeneans per infected host.

**Etymology:** The specific name ‘*sandai*’ honours the ichthyologist Dr Radek Šanda from the Czech National Museum (Prague, Czech Republic) in recognition of his crucial contribution to parasitological work and his precious help in identifying cyprinoid fish hosts during the field trips.

**ZooBank registration (LSID):** urn:lsid:zoobank.org:act:F7B361CD-A677-4135-86CF-0F9A948F9486.

**Description:** (Dimensions of the hard structures are given in Table 3.) Haptor subcircular; tips of inner roots with a hat-like cover; base may show groove-like folds; root long; shaft slightly bowed; point curved and elongated. Ventral bar with blunt, well-developed bilateral processes extending out of bar; median part may show a hollow; membrane elongated, oval, almost 2/3 length of hamuli shaft, with striations ending posteriorly in a median ridge. Dorsal bar straight, may show bifurcated projections near extremities, with attenuated ends inserted into terminal plates. Marginal hooks with globose heel, finger-like toe curved downward, conspicuous shelf, curved point, sickle proper approximately perpendicular to base, gently curved downward to a point slightly exceeding the toe; filament loop extending almost 1/3 handle length. MCO not observed.

**Differential diagnosis:** While no parasitological data were available for *C. umbla*, *G. baicalensis* Bogolepova, 1950 and *G. elegans* were previously reported to parasitize *C. macrostomum* from the Middle East (Mhaisen and Abdul-Ameer, 2013; Mhaisen *et al*., 2018). *Gyrodactylus baicalensis* was originally described from euryhaline hosts, namely *Limnocottus godlewskii* (Dybowski, 1874) and *Batrachocottus multiradiatus* Berg, 1907 (both Perciformes, Cottidae) and then reported on *Planiliza abu* (Heckel, 1843) (Iran) (Mugiliformes, Mugilidae) (Kritsky *et al*., 2013; Al-Jawda and Ali, 2020). *Gyrodactylus sandai* n. sp. is easily distinguishable from both *G. elegans* and *G. baicalensis* by the well-developed bilateral processes on its ventral bar, these features either small or missing in *G. elegans* and *G. baicalensis*. Intraspecific variability was observed, where specimens parasitizing *C. macrostomum* exhibited a slightly longer dorsal bar compared to those parasitizing *C. umbla*. *Gyrodactylus sandai* n. sp., isolated herein from Middle Eastern *C. umbla*, is reminiscent of its Palearctic congener *G. katharineri* Malmberg, 1964 known from a wide range of Palearctic cyprinoids (Pugachev *et al*., 2009). Morphological similarities are mainly in (i) the shape of the hamuli base with covered tips and folds, (ii) the bifurcated projections on the dorsal bar and (ii) the posterior median ridge present in the ventral bar membrane of both species. *Gyrodactylus katharineri* is widely distributed in the Palearctic region, which has resulted in significant morphological intraspecific variability (highly variable sizes of the haptoral sclerotized structures; see meristic data in Pugachev *et al*. [2009]). The newly described *G. sandai* n. sp. and *G. katharineri* had been taxonomically separated regarding the endemism of *C. umbla* to Tigris and Euphrates freshwaters (Froese and Pauly, 2023), this supported significant genetic divergence revealed by mean of ITS sequences (*ITS1* [495 bp long], *p-*distance = 15.4%; *5.8S*-*ITS2* (614 bp long), *p-*distance = 2.9% [Matějusová *et al*., 2001*a* and references herein]). *Gyrodactylus sandai* n. sp. and the newly described *G. mhaiseni* n. sp. exhibit a similarly shaped ventral bar with a posterior median ridge. The main difference between these 2 species is the larger size of each haptoral sclerotized structure exhibited by *G. sandai* n. sp. compared to *G. mhaiseni* n. sp. Moreover, *G. sandai* n. sp. and *G. jurajdai* n. sp. both possess hamuli with conspicuous folds and a ventral bar membrane garnished with a median ridge. Compared to *G. mhaiseni* n. sp., *G. sandai* n. sp. showed (i) longer hamuli (100.0–105.1 *μ*m in *G. sandai* n. sp. *vs* 51.7–59.9 *μ*m in *G. mhaiseni* n. sp.), (ii) longer ventral bar (58.5–63.9 *μ*m in *G. sandai* n. sp. *vs* 19.8–25.6 *μ*m in *G. mhaiseni* n. sp.), with (iii) longer bilateral processes (16.6–22.6 *μ*m in *G. sandai* n. sp. *vs* 1.6–2.5 *μ*m in *G. mhaiseni* n. sp.), (iv) longer dorsal bar (47.0–55.0 *μ*m in *G. sandai* n. sp. *vs* 19.9–25 *μ*m in *G. mhaiseni* n. sp.) and finally (v) longer marginal hooks (45.0–46.5 *μ*m in *G. sandai* n. sp. *vs* 23.2–27.4 *μ*m in *G. mhaiseni* n. sp.), with (vi) longer handle (37.4–39–1 *μ*m in *G. sandai* n. sp. *vs* 17.7–21.6 *μ*m in *G. mhaiseni* n. sp.).

#### ***Gyrodactylus satanicus*** **Rahmouni n. sp.** (Fig. 14)

**Type-host:** *Garra rufa* (Heckel, 1843) (Cyprinoidei: Cyprinidae)

**Figure 14.**
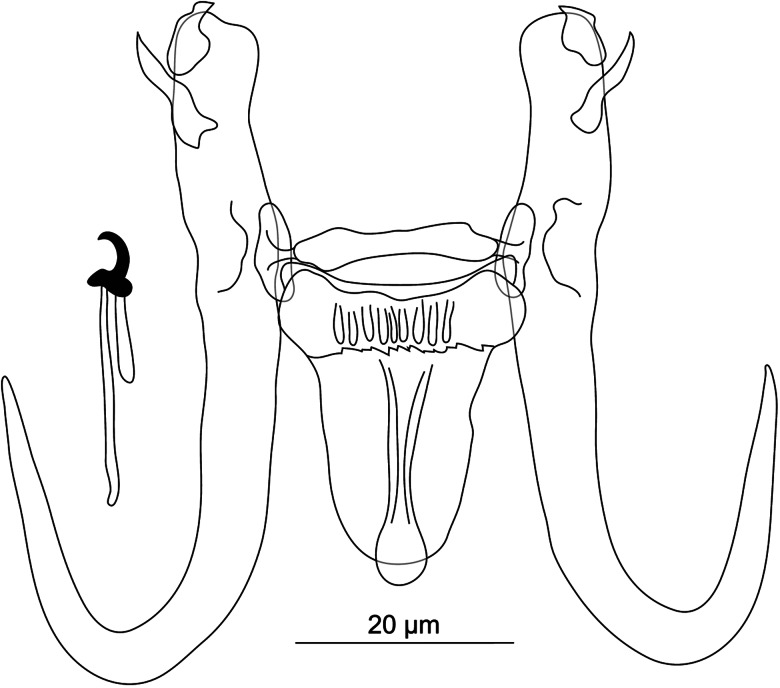
Hard structures of haptor of *Gyrodactylus satanicus* n. sp. ex *Garra rufa*.

**Type-locality:** By the road Sulaymaniyah–Dukan, Little Zab, Iraq

**Site on host**: Fins

**Type material:** Holotype and 2 paratypes (IPCAS M-788).

**Representative DNA sequence:** A nucleotide sequence representing a fragment (1275 bp long; OR773091) including the partial *ITS1* region (683 bp long), *5.8S* rDNA (157 bp) and the partial *ITS2* region (435 bp). No intraspecific variability was found.

**Infection indices:** Prevalence 70%, 1–5 monogeneans per infected host.

**Etymology:** The specific name ‘*satanicus*’ (as an adjective in the nominative singular) refers to the morphology of the hamuli, with horn-like projections reminiscent of a silhouette of the devil's face.

**ZooBank registration (LSID):** urn:lsid:zoobank.org:act:AB3D872E-3A8F-4443-8C33-DBED0892DFE0.

**Description:** (Dimensions of the hard structures are given in Table 3.) Haptor subcircular; tips of inner roots uncovered; base with 2 pairs of horn-like anterior projections and may show groove-like posterior folds; root relatively long, slightly curved inwards; shaft slightly bowed; point curved and elongated. Ventral bar with blunt, short, triangular bilateral processes extending out of bar; median part with hollows; membrane elongated, oval, almost 2/3 length of hamuli shaft ending posteriorly in a median ridge. Dorsal bar straight, slightly constricted at halfway point, with attenuated ends inserted into terminal plates. Marginal hooks with globose heel, blunt toe, conspicuous shelf, curved point, sickle proper gently curved downward to a point perpendicular to toe shelf; filament loop extending almost 1/2 handle length. MCO not observed.

**Differential diagnosis:** *Gyrodactylus elegans* is the sole species hitherto reported from *G. rufa* in Iraq (Mhaisen and Abdul-Ameer, 2013). The typical morphology of the hamuli of *G. satanicus* n. sp. with 2 pairs of horn-like projections makes it easily distinguishable from *G. elegans* and all congeners known so far. The median ridge in the ventral bar membrane exhibited by *G. satanicus* n. sp. is a common feature in Palearctic *Gyrodactylus*, as already discussed above (Pugachev *et al*., 2009). This makes the present study the very first one to report a *Gyrodactylus species* with such features in the Middle East region.

#### ***Gyrodactylus vukicae*** **Rahmouni n. sp.** (Fig. 15)

**Type-host:** *Garra rufa* (Heckel, 1843) (Cyprinoidei: Cyprinidae)

**Figure 15.**
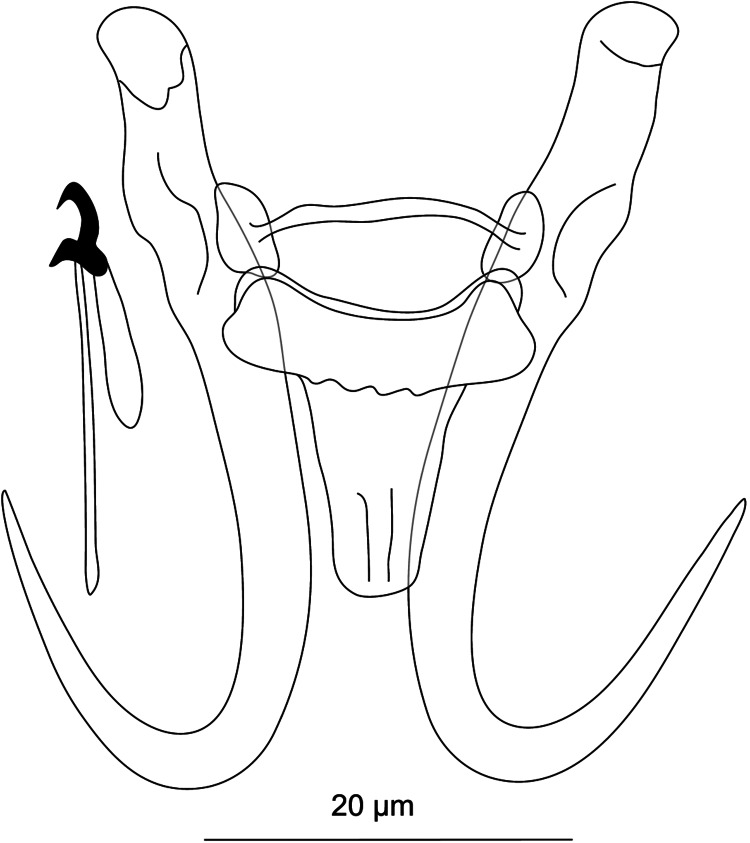
Hard structures of haptor of *Gyrodactylus vukicae* n. sp. ex *Garra rufa*.

**Type-locality:** By the road Sulaymaniyah–Dukan, Little Zab, Iraq

**Site on host:** Fins

**Type material:** Holotype and 1 paratype (IPCAS M-789).

**Representative DNA sequence:** A nucleotide sequence representing a fragment (1218 bp long; OR773090) including the partial *ITS1* region (623 bp), *5.8S* rDNA (157 bp) and the *ITS2* region (438 bp). Minor intraspecific genetic variability was found in the *ITS1* (*P*-distance = 0.4%).

**Infection indices:** Prevalence 20%, 1 monogenean per infected host.

**Etymology:** The specific name ‘*vukicae*’ honours the ichthyologist Dr Jasna Vukić from the Faculty of Sciences, Charles University (Prague, Czech Republic) in recognition of her crucial contribution to parasitological work and her precious help in identifying the cyprinoid hosts during the field trips.

**ZooBank registration (LSID):** urn:lsid:zoobank.org:act:3D2F7DB6-34A1-4534-B7A7-62AD66D14A0C.

**Description:** (Dimensions of the hard structures are given in Table 3.) Haptor subcircular; tips of inner roots with a hat-like cover; base with posterior folds; root long; shaft slightly bowed; point curved and elongated. Ventral bar with blunt, short bilateral processes extending out of bar; median part with no visible hollows; membrane slightly trapezoid, almost 1/3 length of hamuli shaft, may show a posterior ridge. Dorsal bar gently curved, with attenuated ends inserted into terminal plates. Marginal hooks with globose downward heel, triangular, curved finger-like toe curved downward, conspicuous shelf, curved point, sickle proper gently curved downward to a point slightly exceeding toe shelf; filament loop (lamella) extending over 1/2 handle length, MCO not observed.

**Differential diagnosis:** In association with *G. satanicus* n. sp. described above, *G. rufa* was shown to host an additional species described herein as *G. vukicae* n. sp. (Fig. 13). These 2 species are largely discriminated from each other by the atypical pairs of horn-like projections on the hamuli exhibited by *G. satanicus* n. sp. (see above). *Gyrodactylus vukicae* n. sp. is further easily differentiated from *G. elegans*, the only species previously reported from *G. rufa* (Mhaisen and Abdul-Ameer, 2013), by the shape of the ventral bar parts, mainly the blunt short bilateral processes and trapezoid ventral bar membrane in *G. vukicae* n. sp., which are in contrast to the form of the ventral bar in *G. elegans*, which lacks processes and exhibits a long and narrow membrane (Malmberg, 1970; Pugachev *et al*., 2009).

## Discussion

Although the monogenean fauna of freshwater fishes has been extensively studied in Iraq (e.g. Asmar *et al*., 1999; Al-Awadi, 2003; Balasem *et al*., 2003; Mhaisen *et al*., 2003, 2015; Abdullah and Mhaisen, 2005; and numerous local student theses), the present study indicates that only a fraction of its real regional species diversity has been previously uncovered. Nowadays, an integrative approach combining morphological and molecular data is commonly applied in studies on monogeneans. It appears that the diversity of freshwater fish monogeneans revealed by this approach is much higher (Kmentová *et al*., 2016; Dos Santos and Avenant-Oldewage, 2020; Řehulková *et al*., 2020, 2021; Šimková *et al*., 2022) than previously expected on the basis of morphology alone. In the current study, we recorded a total of 33 monogenean species on endemic cyprinoids in Iraq. Comparing the ectoparasitic monogeneans to endoparasitic helminth taxa parasitizing cyprinoids in Iraq, the former appear to be much more diversified (Mhaisen and Abdullah, 2017; Öktener, 2021), similarly as was recorded for cyprinoids in Europe (e.g. Loot *et al*., 2007; Seifertová *et al*., 2008; Rohlenová *et al*., 2011; Krasnovyd *et al*., 2017; Pakosta *et al*., 2018; Benovics *et al*., 2021*b*; see also review of Kuchta *et al*., 2020). This is most likely connected to the overall level of host specificity, which is significantly higher in monogeneans than in, for example, cestodes or digeneans (Rohde, 1979; Whittington *et al*., 2000; Cribb *et al*., 2002; Tkach *et al*., 2003; Kuchta *et al*., 2020). In the present study, we recorded 27 monogenean species only from a single host species, although not all of them can be considered as strictly host-specific, as they were previously recorded also on other cyprinoid hosts in the region (e.g. *D. goktschaicus* Gussev, 1966 is a common parasite of *Barbus* spp. and *Luciobarbus* spp. in the Middle East [Pazooki *et al*., 2006; Koyun *et al*., 2015; Koyuncu *et al*., 2021; Benovics *et al*., 2021*a*], and *D. vistulae* is the *Dactylogyrus* species with the widest distribution range in the western Palearctic [Seifertová *et al*., 2008; Benovics *et al*., 2018, 2023]). One out of these recorded monogenean species are the first record for Iraq, and additional 17 are newly discovered species (4 *Dactylogyrus*, 12 *Gyrodactylus* and 1 *Dogielius*). From the previously described species, only *D. holciki*, found in our study on *A. sellal* and non-described *Alburnus* sp. from the Tabin River and Zalm Stream (near Grdi Go), respectively, was a new host record for Iraq. Although *D. holciki* is a common parasite of *A. mossulensis* Heckel, 1843 and *A. chalcoides* Güldenstädt, 1772 in the Middle East (e.g. Al-Samman *et al*., 2006; Aydoğdu *et al*., 2008; Tunç and Koyun, 2018), described in Iran by Molnár and Jalali (1992), it was not previously reported in Iraq. Considering its host range, this species might be recognized as genus specific for *Alburnus*, and we can assume that its distribution will be restricted to that of the fish of this genus in the Middle East.

Following the host specificity levels designed in Šimková *et al*. (2006) and modified in Benovics *et al*. (2021*a*), and including also previous host records for the respective species, all collected monogenean species fell into 4 categories – true generalists (*D. vistulae*, *D. mokhayeri*, *G. iraqemembranatus* n. sp. and *P. homoion*) parasitizing host species from different cyprinoid families; transitional generalists (*D. goktschaicus*, *D. deziensis* Gussev, Jalali and Molnár, 1993, *D. deziensioides* Gussev, Jalali and Molnár, 1993, *D. molnari*, *D.* cf. *persicus* and *G. sandai* n. sp.) parasitizing hosts belonging to a single subfamily; intermediate specialists (*D. holciki* and *D. lenkorani*); and strict specialists parasitizing only a single host species (all the remaining collected monogenean species). It appears that the cyprinoids in Iraq are predominantly parasitized by highly host-specific monogenean taxa. These also include taxa which are endemic to the Middle Eastern region (only *D. vistulae* was also previously documented outside the Middle East, e.g. Šimková *et al*., 2004; Seifertová *et al*., 2008; Benovics *et al*., 2018), thus we can assume that they cospeciated with their respective cyprinoid host species over long evolutionary time. While *Dactylogyrus*, *Dogielius* and *Paradiplozoon* are almost exclusively limited to cyprinoid fishes (Gibson *et al*., 1996; Pugachev *et al*., 2009), *Gyrodactylus* is the only reported genus with a host range encompassing also other fish taxa (e.g. gasterosteoids, salmonids, silurids, see Bakke *et al*., 1992, 2007; Harris *et al*., 2004). The differences in host specificity levels are most likely connected to life cycle, as gyrodactylids are strictly viviparous; thus, their dispersal into new hosts is limited to direct contact between fish (Bakke *et al*., 2007) rendering the parasites opportunistic host-switchers. Surprisingly, only *G. iraqemembranatus* n. sp. and *G. sandai* n. sp. were collected from more than 1 species within our study; nevertheless, 2 host species for each of these *Gyrodactylus* spp. were collected from the same site (Kani Shok and wadi Kalat Shirah, respectively). These observations and recorded host–parasite associations only support the low host preferences of *Gyrodactylus* parasites. On the other hand, *Dactylogyrus*, *Dogielius* and *Paradiplozoon* are oviparous monogenean taxa which are known to have developed various morphological (Sasal *et al*., 1999; Huyse and Volckaert, 2002; Šimková *et al*., 2006) and ecological (Whittington *et al*., 1999; Buchmann and Lindenstrøm, 2002; Whittington and Kearn, 2011) adaptations to seek and infest associated hosts and the ones most suitable for retaining parasite populations. These adaptations putatively limit the host-switching capacities of the given monogenean species and therefore their dispersal capacity is restricted to the dispersal of their respective hosts. The excepted high host specificity among dactylogrid monogeneans was in accordance with our observations, as the majority of species within the genera *Dactylogyrus* and *Dogielius* are restricted either to a single host species, or several local congeners. In Europe and North America, 30 and 61% of *Dactylogyrus* species, respectively, appear to be strictly host specific (Kuchta *et al*., 2020). In our local study, focusing only on monogenean diversity in Iraq, 15 out of 16 *Dactylogyrus* species were recorded only from a single host species (although several species exhibit wider host range, as is discussed above). A similar high level of host specificity could initially be assumed also for *Dogielius* species, as *D. mokhayeri* was initially reported only from *Leuciscus vorax* (Heckel, 1843), an endemic species in the Middle East (Jalali and Molnár, 1990; Abdullah and Mhaisen, 2005); however, our records suggest that the host range for this monogenean species might also encompass other leuciscid species in the Middle East, and thus this species is endemic to the Middle East without any host preferences. The other 2 previously described *Dogielius* species (i.e. *D.* cf. *persicus* and *D. molnari* Jalali, 1992) were reported in Iraq for the first time by Abdullah and Mhaisen (2005), who recorded all 3 *Dogielius* species from 3 cyprinoid species in the Grater Zab River. In addition, it appears that in the Tabin River basin a potentially new *Dogielius* species can also be found, as the previously undescribed species was collected from *C. umbla* in Wadi Kalat Shirah valley. This putatively new *Dogielius* species will be properly described after the collection of additional material for morphometric data, and the description will be included in a study also investigating congeners from other geographical regions.

The phylogenetic analyses performed on the species of 2 selected monogenean genera (*Dactylogyrus* and *Gyrodactylus*) revealed that the endemic congeneric species from Iraq did not form a monophyletic group. The majority of *Dactylogyrus* species studied herein were positioned within the phylogenetic lineage A, encompassing Middle Eastern, European, North African and North American species. Several well-supported groups were formed within lineage A; however, the molecular data used in this study were not sufficiently phylogenetically informative to fully resolve the relationships between them. Within the groups, clustered species shared the morphotypes of taxonomically important characters, mainly the hard parts of the attachment organ. *Dactylogyrus* species associated with cyprinid hosts (i.e. *Barbus*, *Capoeta*, *Carasobarbus*, *Cyprinion*, *Garra*, *Luciobarbus* and *Paracapoeta*) were in paraphyly and were included in the 5 phylogenetic groups. While *D. persis* and *D. barbuli* clustered together with north African, Middle Eastern and European (Iberian in this case) congeners possessing the ‘carpathicus’ morphotype of haptoral connective ventral bar (see Pugachev *et al*. [2009] for classification of morphotypes), *D. goktschaicus*, *D. deziensis* and *D. deziensioides* formed a group with European and Middle Eastern congeners. The latter group possesses the ventral bar of various shapes (especially the ‘tissensis’ and ‘rutili’ type); however, it is considered as ‘less complex’, i.e. with a lower number of extremities, when compared to the most complex ventral bar (Benovics *et al*., 2021*a*). The grouping of *Dactylogyrus* spp. of cyprinids within lineage A is in accordance with the proposed dual historical divergence and dispersion patterns for cyprinids (Doadrio, 1990; Casal-Lopéz and Doadrio, 2018) and their associated parasites (Benovics *et al*., 2021*a*). By the distribution of the respective *Dactylogyrus* species from Iraqi cyprinoids within 2 morphogroups, we can hypothesize that they historically split within the Middle Eastern region and while the group encompassing *D. barbuli* and phylogenetically close congeners sharing the ‘carpathicus’ morphotype of ventral bar dispersed *via* North Africa, the other species dispersed directly into Europe *via* the Balkan-Anatolian connection (Gomphoterium Land Bridge, Steininger and Rögl [1984]; Perea *et al*. [2010]). Unprecedented (see Šimková *et al*. [2004]; Benovics *et al*. [2021*a*] or Acosta *et al*. [2022] for comparison) is the phylogenetic position of the 2 presumably host-specific *Dactylogyrus* species, *D. microcirrus* and *D. macrosotomi* (from *P. trutta* and *C. macrostomum*, respectively), which formed an individual phylogenetic lineage. The deep nodal split of this lineage from other *Dactylogyrus* lineages and the inclusion of the Iraqi cyprinoid-specific *Dactylogyrus* species within suggest early diversification in the region, and we can assume that this lineage is endemic to the Middle Eastern area. We can hypothesize that this lineage may include also other endemic congeners sharing similar morphological features (e.g. *D. barbioides* Gussev *et al*., 1993, described from *Arabibarbus grypus* [Heckel, 1843] or *D. cyprinioni* parasitizing *C. macrostomum*, see Pugachev *et al*. [2009]); however, the molecular data for these species are still missing. The ancestral origin of this lineage in the Middle East can be linked to the phylogenetic relationships of their hosts, as *Paracapoeta* is a sister to endemic *Capoeta*, with the basal position (Turan *et al*., 2022), and *Cyprinion* represents a divergent phylogenetic entity in the region (Durand *et al*., 2002; Yang *et al*., 2015) (although, the phylogenetic relationships among the *Cyprinion* species are not yet well investigated). *Dactylogyrus cyprinioni* is presumably specific to *Cyprinion* spp. which further supports our assumption of the position of this *Dactylogyrus* species within the endemic lineage; however, further investigation is required.

*Dactylogyrus regius* n. sp. formed a well-supported group with congeners possessing the inverted T-shape morphotype of ventral bar, and the ‘chondrostomi’ morphotype of MCO, which are common for *Dactylogyrus* species parasitizing *Chondrostoma s. l.* hosts, such as *D. ergensi*, *D. elegantis* and *D. globulatus* (Pugachev *et al*., 2009; Řehulková *et al*., 2020; Benovics *et al*., 2020*a*, 2021*b*). Therefore, we can assume that the species of this group are historically associated with *Chondrostoma* hosts, and only secondarily host-switched and speciated on other leuciscids (e.g. *D. tissensis* is a common species of *Alburnoides* spp. [Benovics *et al*., 2018], and *D. sagittarius* parasitizes *Telestes* [Benovics *et al*., 2021*b*]; however, all these species share the same morphological features). Contrastingly, *Dactylogyrus anoigeus* n. sp. described from *A. marmid* was not phylogenetically close to congeners associated with *Abramis* (e.g. *D. auriculatus* [von Nordmann, 1832] and *D. zandti* Bychowsky, 1933 [Dzika, 2002; Krasnovyd *et al*., 2020; Dedić *et al*., 2023]) or *Blicca* (e.g. *D. cornu* Linstow, 1878 [Soylu, 2012; Krasnovyd *et al*., 2020], despite the phylogenetic proximity of these host genera [Teimori *et al*., 2015; Schönhuth *et al*., 2018]). *Dactylogyrus anoigeus* n. sp. grouped with congeners parasitizing *Squalius* spp., such as *D. folkmanovae*, *D. vranoviensis* (Seifertová *et al*., 2008; Benovics *et al*., 2023) and the herein described *D. rivalis* n.sp., which appears to be a species endemic to the Middle East.

Novel molecular data for 2 *Dactylogyrus* species parasitizing *G. rufa* (i.e. *D. acinacus* and *D. medicus* n. sp.) revealed the phylogenetic proximity of these species to congeners with a putative origin in Eastern Asia, and associated with the cyprinids *Carassius* and *Cyprinus* (e.g. *D. anchoratus* [Dujardin, 1845], *D. formosus* Kulwiec, 1927 and *D. vastator* [Nybelin, 1924]), *Barbonymus* (*D. tapienensis* and *D. viticulus*), Middle Eastern *Capoeta* (i.e. *D. pulcher*) and North African *Carasobarbus* (i.e. *D. marocanus*). Benovics *et al*. (2021*a*) previously discussed the phylogenetic relationships of the species within lineage C; however, these new findings might finally resolve the origin and dispersal of *Dactylogyrus* species into Africa. Cyprinids of the genus *Garra* can be found throughout Southwest Asia with a distribution range extending from the Indus River up to the Nile basin and Ethiopia (Stiassny and Getahun, 2007). The distribution and phylogeography of the genus might suggest that *Garra* species served as the historical mediator for the dispersion of *Dactylogyrus* between Eastern Asia and Africa. By similarities in their morphology, we can expect that other specialists of *Garra* (e.g. *D. rectotrabus*) will also belong to this phylogenetic lineage; however, additional sequences of *Dactylogyrus* parasitizing African and Asian *Garra* are required to test this hypothesis.

Similarly to *Dactylogyrus*, *Gyrodactylus* species in Iraq did not form a monophyletic group. Overall, the gyrodactylid species studied herein clustered in 3 distinct lineages of different origins, i.e. Palearctic and African. *Gyrodactylus vukicae* n. sp. and *G. satanicus* n. sp. found to parasitize *G. rufa* were grouped within lineage A, whilst *G. satanicus* n. sp. was genetically closer to *G. hildae* from African *O. niloticus* than to its congener from the same host. Well-supported monophyly was found for *G. mhaiseni* n. sp., *Gyrodactylus sandai* n. sp. and the undescribed *Gyrodactylus* sp. 3 and *Gyrodactylus* sp. 4 collected from *A. sellal* and *B. lacerta*, respectively. Specifically, the sister position of *G. mhaiseni* n. sp. to *Gyrodactylus* sp. 3 might suggest that these species are strongly associated with their *Alburnus* hosts in the Middle East and might represent a case of intrahost duplication on *A. sellal*. Previously, Huyse and Volckaert (2005) revealed that *Gyrodactylus* species parasitizing the gills originated from host-switch and that only less host-specific fin *Gyrodactylus* co-speciated with their goby hosts, indicating that in their host–parasite system cospeciation is not associated with high host specificity in viviparous parasites. Moreover, they showed that the host switching of *Gyrodactylus* from *Gasterosteus aculeatus* Linnaeus, 1758 to non-congeneric fish hosts most likely facilitated the adaptive radiation of numerous highly host-specific *Gyrodactylus* species. According to Hahn *et al*. (2015), cophylogenetic patterns are trackable also at the population level of *Gyrodactylus* parasites of *G. aculeatus*. Their study also supported host-switch as a common event in the evolutionary history of *Gyrodactylus*. In our case, we can expect that intrahost duplication will play an important role in the speciation of *Gyrodactylus* in geographically isolated regions, just as in *Thaparocleidus* (Šimková *et al*., 2013) and *Cichlidogyrus* (Mendlová *et al*., 2012). The distribution of *A. sellal* is rather widespread in the Middle East, as this species can be found in the rivers of the Mediterranean and Red Sea basins (e.g. Bogutskaya, 1997; Kuru, 2004; Dağlı and Erdemli, 2009; Erk'akan and Özdemir, 2011; Bi̇reci̇kli̇gi̇l et al., 2016), where we can expect that different monogenean species evolved in the individual parapatric populations independently. *Alburnus sellal* was also parasitized by *G. iraqemembranatus* n. sp., a highly genetically and morphologically divergent *Gyrodactylus* species. This species was recorded on 3 phylogenetically non-congeneric host species, all collected in the Tabin River basin (however, at different collection sites) and represents a phylogenetically divergent, basally positioned, lineage. We can only assume that other endemic Middle Eastern *Gyrodactylus* spp. will share the basal position with *G. iraqemembranatus* n. sp.; however, molecular data on local species are still scarce. The 3 distinct lineages of the analysed Iraqi *Gyrodactylus* revealed by genetic data are, in fact, in accordance with the haptoral morphology of the gyrodactylid species, we studied rather than with host phylogeny, similar pattern as was observed in African *Cichlidogyrus* (Rahmouni *et al*., 2022), or in Nearctic *Dactylogyrus* (Šimková *et al*., 2006). Except for the morphologically unidentified species included in the phylogenetic analyses, all *Gyrodactylus* belonging to lineage A exhibited a similar morphotype of ventral bar, mainly characterized by the presence of lateral processes, a feature lacking in *G. blazeki* n. sp. and their congeners of lineage D, as well as in *G. iraqemembranatus* n. sp., which was positioned as the most basal lineage F. Except for *G. vukicae* n. sp., the species of lineage A identified in this study on endemic cyprinoids from Iraq showed ventral bars with a ridge in the median part of the membrane, a well-known characteristic of Eurasian gyrodactylid lineages, identified as the *G. katharineri* group in Malmberg (1970) – based on records of *G. katharineri* from a wide range of cyprinoids in this region (Pugachev *et al*., 2009). In this study, *G. sandai* n. sp. was discriminated from *G. katharineri* using DNA sequences following Huyse *et al*. (2003) and Ziȩtara and Lumme (2003), while no haptoral features were found to morphologically differentiate between these 2 species. This may indicate a cryptic speciation as previously found in the Nearctic system (Rahmouni *et al*., 2023). Finally, *G. iraqemembranatus* n. sp. possesses a ‘simple’ form of haptoral sclerites, with a ventral bar lacking both lateral processes and a membrane, although it possesses the typical shape of marginal hooks, especially with prolonged basal part. Therefore, we can hypothesize that *Gyrodactylus* species with such features (see the diagnosis section for *G. iraqemembranatus* n. sp.) form a basal clade together with *G. iraqemembranatus* n. sp.; however, their molecular data are required for future phylogenetic studies.

## Supporting information

Benovics et al. supplementary material 1 Benovics et al. supplementary material

Benovics et al. supplementary material 2 Benovics et al. supplementary material

## Data Availability

The data supporting the conclusions of this study are included in this article. The type-material of the new species described in this study was deposited in the Helminthological Collection of the Institute of Parasitology, Czech Academy of Sciences, České Budĕjovice, Czech Republic under the accession number IPCAS M-782 – 793. The newly generated sequences were submitted to the GenBank database under accession numbers OR773082 – OR773094, OR817699 – OR817715, and OR817682 – OR817698.

## Supplementary material

The supplementary material for this article can be found at https://doi.org/10.1017/S0031182023001348
