# Supplementary material for: Uncovering the monogenean species diversity of cyprinoid fish in Iraq using an integrative approach
Source: Parasitology. 2023 Dec 20;151(2):220–46. doi: 10.1017/S0031182023001348 (PMC10941050; doi:10.1017/S0031182023001348)
Supplement: Benovics et al. supplementary material 1 — Benovics et al. supplementary material [file S0031182023001348sup001.pdf]

Supplementary file 1: Tables comprising metadata and sequence accession numbers for each host species and monogenean species included in phylogenetic analyses.

Supplementary Table 1. List of host species with the respective GenBank accession number for a representative cytochrome *b* sequence per each population.

Fish species	Locality	Accession number cyt b
Acanthobrama marmid	Du Choman, Aw-e Shiler River	OQ797988
Alburnus sellal	wadi Kalat Shirah, tributary of Tabin River	OQ797989
	Zahrzi, Tabin River	OQ797989
	Grdi Go, Zalm Stream	OQ797991
Alburnus sp.	Grdi Go, Zalm Stream	OQ797992
Barbus lacerta	Kani Shok, tributary of Tabin River	OQ797993
Carasobarbus luteus	Zahrzi, Tabin River	KU524920
Carasobarbus luteus	Grdi Go, Zalm Stream	AF145944
Paracapoeta trutta	Kani Shok, tributary of Tabin River	JF798333
Capoeta umbla	wadi Kalat Shirah, tributary of Tabin River	KY065268
Cyprinion macrostomum	wadi Kalat Shirah, tributary of Tabin River	OQ797995
Garra rufa	by the road Suleymania-Dukan, Little Zab	OQ797996
Chondrostoma regium	Du Choman, Aw-e Shiler River	OQ797997
Luciobarbus barbulus	Du Choman, Aw-e Shiler River	OQ797998
Squalius berak	Kani Shok, tributary of Tabin River	OQ797999
Squalius lepidus	Du Choman, Aw-e Shiler River	OP728025

Newly generated sequences are marked by asterisks (*).

Supplementary Table 2. List of *Dactylogyrus* and *Ancyrocephalus* species used in the phylogenetic analyses with GenBank accession numbers for the sequences of two ribosomal regions.

Dactylogyrus species	Host species	Country	Accession number 18S+ITS1	Accession number 28S
Dactylogyrus acinacus	*Garra rufa*	Iraq	OR817711*	OR817696*
Dactylogyrus affinis	*Barbus cyri*	Iran	MZ031066	MZ031054
Dactylogyrus alatus	*Alburnus neretvae*	Bosnia and Herzegovina	MG792842	MG792956
Dactylogyrus andalousiensis	*Luciobarbus comizo*	Spain	MN365672	MN338207
Dactylogyrus anchoratus	*Carassius gibelio*	Croatia	KY859795	KY863555
Dactylogyrus anoigeus n. sp.	*Acanthobrama marmid*	Iraq	OR817699*	OR817682*
Dactylogyrus arcus	*Luxilus chrysocephalus*	Arkansas	OM108553	OM108517
Dactylogyrus atlasensis	*Luciobarbus lepineyi*	Morocco	KY629337	KY629356
Dactylogyrus atromaculatus	*Semotilus atromaculatus*	New York	OM108555	OM108519
Dactylogyrus attenuatus	*Semotilus atromaculatus*	Wisconsin	OM108556	OM108520
Dactylogyrus auriculatus	*Abramis brama*	Czech Republic	MG792838	MG792952
Dactylogyrus aviunguis	*Nocomis biguttatus*	Wisconsin	OM108557	OM108521
Dactylogyrus balistae	*Luciobarbus bocageii*	Portugal	KY629344	MN338205
Dactylogyrus balkanicus	*Barbus prespensis*	Greece	KY201093	KY201107
Dactylogyrus barbuli	*Luciobarbus barbulus*	Iraq	OR817712*	OR817692*
Dactylogyrus barbuli	*Luciobarbus xanthopterus*	Iraq	MZ031076	MZ031065
Dactylogyrus behoussai	*Luciobarbus moulouyensis*	Morocco	KX578025	KX553862
Dactylogyrus bifurcatus	*Pimephales notatus*	Arkansas	OM108558	OM108522
Dactylogyrus bocageii	*Luciobarbus bocageii*	Portugal	MN365671	KY629347
Dactylogyrus borealis	*Phoxinus* sp.	Bosnia and Herzegovina	KY629343	KY629372
Dactylogyrus borjensis	*Luciobarbus zayanensis*	Morocco	MN974257	MN973819
Dactylogyrus carassobarbi	*Carasobarbus luteus*	Iraq	MZ031071	MZ031060
Dactylogyrus caucasicus	*Alburnus devolii*	Albania	MG792840	MG792954
Dactylogyrus cf. boopsi	*Pimephales notatus*	Arkansas	OM108561	OM108525
Dactylogyrus cf. chrosomi	*Chrosomus neogaeus*	Wisconsin	OM108562	OM108526
Dactylogyrus cf. parvicirrus	*Notemigonus crysoleucas*	New York	OM108563	OM108527
Dactylogyrus confusus	*Clinostomus elongatus*	Wisconsin	OM108565	OM108529
Dactylogyrus conchatus	*Telestes muticellus*	Italy	MW443036	MW443033
Dactylogyrus cornu	*Vimba vimba*	Czech Republic	KY629342	KY629371
Dactylogyrus crivellius	*Barbus prespensis*	Greece	KY201094	KY201108
Dactylogyrus deziensioides	*Luciobarbus barbulus*	Iraq	OR817709*	OR817694*
Dactylogyrus deziensis	*Luciobarbus barbulus*	Iraq	OR817713*	OR817695*
Dactylogyrus dirigerus	*Chondrostoma vardarense*	Greece	MG792876	MG792992
Dactylogyrus draaensis	*Luciobarbus lepineyi*	Morocco	MN974258	MN973816
Dactylogyrus dyki	*Barbus cyclolepis*	Greece	MG792856	MG792971
Dactylogyrus ergensi	*Chondrostoma vardarense*	Greece	MG792878	MG792993
Dactylogyrus fallax	*Vimba vimba*	Czech Republic	KY629341	KY629370
Dactylogyrus falsiphallus	*Luciobarbus massaensis*	Morocco	KX578024	KX553861
Dactylogyrus fimbriphallus	*Luciobarbus lepineyi*	Morocco	KY629332	KY629357
Dactylogyrus flagristylus	*Nocomis biguttatus*	Wisconsin	OM108566	OM108530
Dactylogyrus folkmanovae	*Squalius cephalus*	Croatia	MG792911	MG793028

Dactylogyrus formosus	*Carassius gibelio*	Croatia	MG792869	MG792984
Dactylogyrus globulatus	*Chondrostoma soetta*	Italy	MW443035	MW443032
Dactylogyrus goktschaicus	*Barbus cyri*	Iran	MZ031067	MZ031055
Dactylogyrus goktschaicus	*Barbus lacerta*	Iraq	OR817701*	OR817684*
Dactylogyrus gracilis	*Capoeta buhsei*	Iran	MZ031068	MZ031056
Dactylogyrus guadianensis	*Luciobarbus comizo*	Spain	MN365674	MN338209
Dactylogyrus holciki	*Alburnus* sp.	Iraq	OR817700*	OR817683*
Dactylogyrus cheloideus	*Rhinichthys atratulus*	Wisconsin	OM108567	OM108531
Dactylogyrus inutilis	*Capoeta trutta*	Iraq	OR817704*	OR817687*
Dactylogyrus ksibii	*Luciobarbus ksibi*	Morocco	MN974252	MN973812
Dactylogyrus kulindrii	*Carasobarbus fritschii*	Morocco	KY629336	KY629354
Dactylogyrus kulwieci	*Luciobarbus xanthopterus*	Iraq	MZ031075	MZ031064
Dactylogyrus lachneri	*Nocomis biguttatus*	Wisconsin	OM108568	OM108532
Dactylogyrus legionensis	*Luciobarbus graellsii*	Spain	MN365678	MN338210
Dactylogyrus lenkorani	*Capoeta buhsei*	Iran	MZ031069	MZ031057
Dactylogyrus lenkorani	*Capoeta umbla*	Iraq	OR817706*	OR817689*
Dactylogyrus lenkoranoïdes	*Luciobarbus graellsii*	Spain	MN365676	MN338211
Dactylogyrus leptus	*Chondrostoma knerii*	Croatia	MG792871	MG792986
Dactylogyrus linstowi	*Luciobarbus capito*	Iran	MZ031073	MZ031062
Dactylogyrus linstowoïdes	*Luciobarbus guiraonis*	Spain	KY629329	KY629349
Dactylogyrus macrostomi	*Cyprinion macrostomum*	Iraq	OR817708*	OR817690*
Dactylogyrus marocanus	*Carasobarbus fritschii*	Morocco	KY629333	KY629355
Dactylogyrus martinovici	*Pachychilon pictum*	Greece	MG792885	MG793001
Dactylogyrus mascomai	*Luciobarbus graellsii*	Spain	MN365680	MN338215
Dactylogyrus medicus n. sp.	*Garra rufa*	Iraq	OR817710*	OR817691*
Dactylogyrus microcirrus	*Paracapoeta trutta*	Iraq	OR817705*	OR817688*
Dactylogyrus nanoides	*Squalius prespensis*	Greece	MG792923	MG793045
Dactylogyrus nanus	*Rutilus rubilio*	Italy	MK434933	MK434953
Dactylogyrus octopus	*Tropidophoxinellus spartiaticus*	Greece	MG792950	MG793065
Dactylogyrus omenti	*Aulopyge huegelii*	Bosnia and Herzegovina	KY201091	KY201105
Dactylogyrus opertus	*Telestes muticellus*	Italy	MK434944	MK434964
Dactylogyrus opsopoeodi	*Opsopoeodus emiliae*	Mississippi	OM108569	OM108533
Dactylogyrus pectenatus	*Pimephales promelas*	Wisconsin	OM108571	OM108535
Dactylogyrus perlus	*Luxilus chrysocephalus*	Arkansas	OM108572	OM108536
Dactylogyrus persis	*Carasobarbus luteus*	Iraq	OR817702*	OR817685*
Dactylogyrus petenyi	*Barbus cyclolepis*	Greece	MG792857	MG792972
Dactylogyrus petkovici	*Pachychilon pictum*	Greece	MG792887	MG793003
Dactylogyrus polylepidis	*Achondrostoma arcasii*	Spain	MN365664	MN338198
Dactylogyrus pulcher	*Capoeta razii*	Iran	MZ031070	MZ031058
Dactylogyrus recisus	*Pachychilon macedonicum*	Greece	MG792882	MG792998
Dactylogyrus regius n. sp.	*Chondrostoma regium*	Iraq	OR817707*	OR817693*
Dactylogyrus rhinichthius	*Rhinichthys atratulus*	Wisconsin	OM108573	OM108537
Dactylogyrus rivalis n. sp.	*Squalius lepidus*	Iraq	OR817714*	OR817697*
Dactylogyrus rosickyi	*Pachychilon pictum*	Greece	MG792888	MG793004
Dactylogyrus rutili	*Rutilus lacustris*	Greece	MG792900	MG793016
Dactylogyrus rysavyi	*Alburnoides oeconomui*	Greece	MG792851	MG792965
Dactylogyrus sagittarius	*Telestes muticellus*	Italy	MW443037	MW443034
Dactylogyrus sandai	*Telestes kaedicus*	Croatia	MG792942	MG793057
Dactylogyrus scorpius	*Luciobarbus rifensis*	Morocco	KX578023	KX553860
Dactylogyrus sekulovici	*Pachychilon pictum*	Greece	MG792889	MG793005
Dactylogyrus semotilus	*Luxilus chrysocephalus*	Arkansas	OM108574	OM108538
Dactylogyrus sp.	*Luciobarbus xanthopterus*	Iraq	MZ031074	MZ031063
Dactylogyrus sphyrna	*Vimba vimba*	Czech Republic	MG792951	MG793066
Dactylogyrus suecicus	*Rutilus lacustris*	Greece	MG792901	MG793017
Dactylogyrus tapienensis	*Barbonymus schwanenfeldii*	South African Republic	OR081826	OR077124
Dactylogyrus tissensis	*Alburnoides oeconomui*	Greece	MG792852	MG792966
Dactylogyrus varius	*Luciobarbus massaensis*	Morocco	KX578026	KX553863
Dactylogyrus vastator	*Carassius gibelio*	Croatia	KY207446	KY629366
Dactylogyrus venusti	*Cyprinella venusta*	Mississippi	OM108588	OM108552
Dactylogyrus vistulae	*Squalius lepidus*	Iraq	OR817715*	OR817698*
Dactylogyrus vistulae	*Squalius prespensis*	Greece	MG792925	MG793043
Dactylogyrus viticulus	*Barbonymus schwanenfeldii*	South African Republic	OR081827	OR077125
Dactylogyrus volutus	*Carasobarbus fritschii*	Morocco	KY629334	KY629353
Dactylogyrus vranoviensis	*Squalius squalus*	Croatia	MG792931	MG793048
Dactylogyrus vukicae	*Delminichthys adspersus*	Bosnia and Herzegovina	MG792881	MG792995
Dactylogyrus yinwenyingae	*Squalius lucumonis*	Italy	MK434939	MK434959
Dactylogyrus zandti	*Abramis brama*	Czech Republic	MG792839	MG792953
Dactylogyrus zatensis	*Carasobarbus fritschii*	Morocco	KY629335	KY629352
Ancyrocephalus percae	*Perca fluviatilis*	Finland	AJ490166	KF499080

The list includes also host species and countries of collection for each *Dactylogyrus* species. Newly-generated sequences are marked by asterisks (*).

Supplementary Table 3. List of *Gyrodactylus* and *Macrogyrodactylus* species used in the phylogenetic analyses with GenBank accession numbers for the sequences of one ribosomal region.

***Gyrodactylus* species**	**Host species**	**Country**	**Accession number**
Gyrodactylus alekosi	*Clarias gariepinus*	Mozambique	FR850682
Gyrodactylus arcuatus	N/A	N/A	AF156668
Gyrodactylus azeezsaeedi n. sp.	*Squalius berak*	Iraq	OR773093*
Gyrodactylus blazeki n. sp.	*Alburnus* sp.	Iraq	OR773085*
Gyrodactylus botnicus	*Phoxinus phoxinus*	Finland	AF484542
Gyrodactylus branchicus	*Gasterosteus aculeatus*	Belgium	AF156669
Gyrodactylus bubyri	*Pomatoschistus microps*	Belgium	AF328868
Gyrodactylus danastriae	*Phoxinus phoxinus*	Poland	HM192925
Gyrodactylus derjavini	N/A	Great Britain	AJ001840
Gyrodactylus derjavinoides	N/A	N/A	AJ132259
Gyrodactylus ergensi	*Sarotherodon galilaeus*	Senegal	FN394985
Gyrodactylus gobii	N/A	Czech Republic	AJ407922
Gyrodactylus hildae	*Oreochromis niloticus*	Ethiopia	FJ231869
Gyrodactylus iraqemembranatus n. sp.	*Paracapoeta trutta*	Iraq	OR773087*
Gyrodactylus jiroveci	*Barbatula barbatula*	Czech Republic	AM502860
Gyrodactylus jurajdai n. sp.	*Chondrostoma regium*	Iraq	OR773088*
Gyrodactylus kobayashii	*Carassius auratus*	China	KJ524572
Gyrodactylus laevis	*Phoxinus phoxinus*	Finland	AY278036
Gyrodactylus laevisoides	*Chrosomus eos*	Canada	KF263527
Gyrodactylus lomi	*Squalius cephalus*	Czech Republic	AJ407929
Gyrodactylus luciopercae	*Perca fluviatilis*	Czech Republic	AJ407931
Gyrodactylus macronychus	*Phoxinus phoxinus*	Czech Republic	AJ407893
Gyrodactylus malalai	*Oreochromis niloticus*	Sudan	FR695484
Gyrodactylus mediotorus	*Notropis hudsonius*	Canada	KF178301
Gyrodactylus mhaiseni n. sp.	*Alburnus sellal*	Iraq	OR773082*
Gyrodactylus mhaiseni n. sp.	*Alburnus sellal*	Iraq	OR773083*
Gyrodactylus nigritae	*Synodontis nigrita*	Senegal	FR850686
Gyrodactylus nipponensis	*Anguilla japonica*	Japan	AB063295
Gyrodactylus nyanzae	*Oreochromis niloticus*	Zimbabwe	LN849939
Gyrodactylus occupatus	*Tilapia* sp.	Zimbabwe	LN849940
Gyrodactylus pannonicus	*Phoxinus phoxinus*	Slovak Republic	HM192915
Gyrodactylus parisellei	*Oreochromis niloticus*	Zimbabwe	LN849941
Gyrodactylus poeciliae	*Poecilia caucana*	Venezuela	AJ001844
Gyrodactylus prostae	*Rutilus rutilus*	Czech Republic	AJ567673
Gyrodactylus pterygialis	N/A	N/A	AJ581657
Gyrodactylus pungitii	*Poecilia caucana*	Venezuela	AJ001845
Gyrodactylus rhodei	*Rhodeus sericeus*	Czech Republic	AJ407933
Gyrodactylus rivularae	*Abbottina rivularis*	China	HM185818
Gyrodactylus rysavyi	*Clarias anguillaris*	Senegal	FR850679
Gyrodactylus salaris	N/A	N/A	AJ001847
Gyrodactylus sandai n. sp.	*Capoeta umbla*	Iraq	OR773089*
Gyrodactylus satanicus n. sp.	*Garra rufa*	Iraq	OR773091*
Gyrodactylus sp. 3	*Luciobarbus barbulus*	Iraq	OR773092*
Gyrodactylus sp. 3	*Alburnus sellal*	Iraq	OR773084*
Gyrodactylus sp. 4	*Barbus lacerta*	Iraq	OR773086*
Gyrodactylus sturmbaueri	*Psedocrenilabrus philander*	Zimbabwe	LN849938
Gyrodactylus synodonti	*Synodontis nigrita*	Senegal	FR850684
Gyrodactylus ulinganisus	*Oreochromis mossambicus*	South African Republic	FJ231870
Gyrodactylus vimbi	*Squalius cephalus*	Czech Republic	AJ407936
Gyrodactylus vukicae n. sp.	*Garra rufa*	Iraq	OR773090*
Gyrodactylus vukicae n. sp.	*Garra rufa*	Iraq	OR773094*
Macrogyrodactylus karibae	*Clarias gariepinus*	Zimbabwe	GU252715

The list includes also host species and countries of collection for each *Gyrodactylus* species. Newly-generated sequences are marked by asterisks (*). N/A = data were not available in GenBank database.
