# Supplementary material for: Uncovering the monogenean species diversity of cyprinoid fish in Iraq using an integrative approach
Source: Parasitology. 2023 Dec 20;151(2):220–46. doi: 10.1017/S0031182023001348 (PMC10941050; doi:10.1017/S0031182023001348)

Supplementary file 2: Microphotographs of collected monogenean species

Fig. S1 ***Dactylogyrus anoigeus*** **n. sp. ex** ***Acanthobrama marmid*****. Microphotograph of (a) the haptoral structures and (b) the male copulatory organ and vagina.**

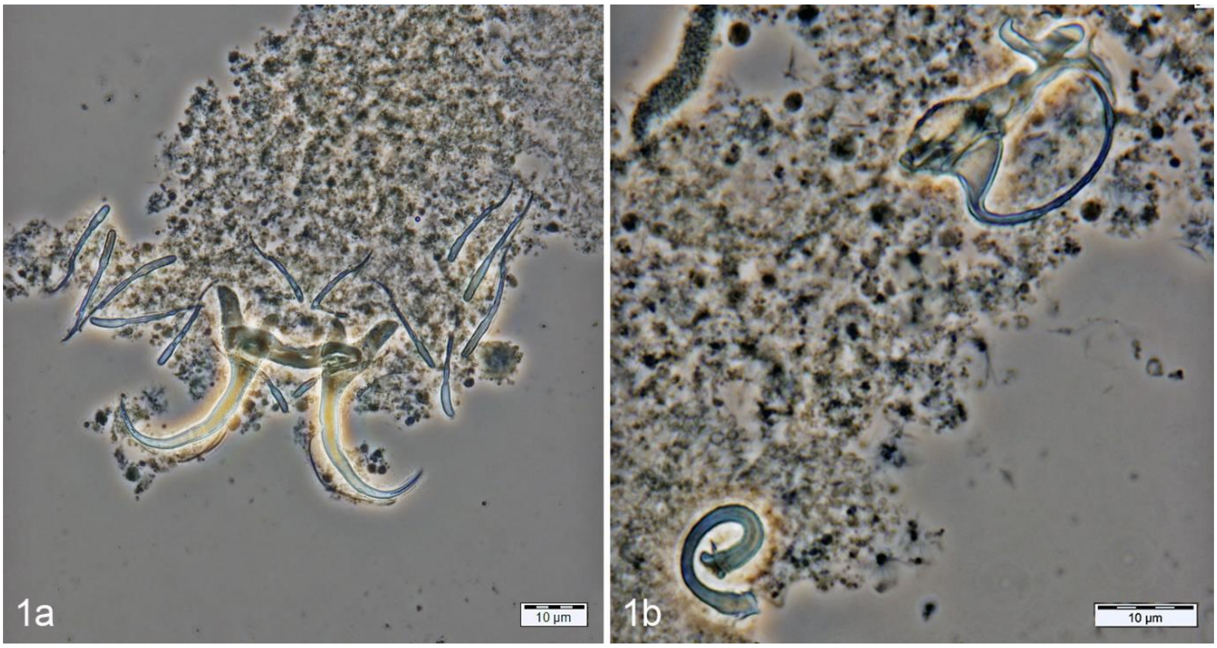

Fig. S2 ***Dactylogyrus medicus*** **n. sp. ex** ***Garra rufa*****. Microphotograph of (a) the haptoral structures and (b) the male copulatory organ.**

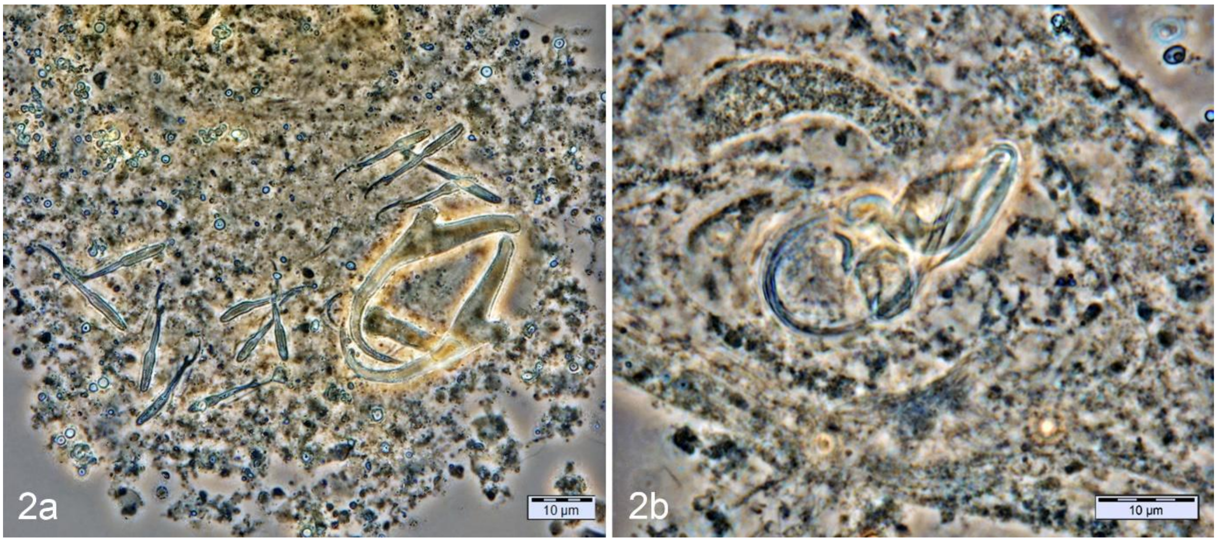

Fig. S3 ***Dactylogyrus regius*** **n. sp. ex** ***Chondrostoma regium*****. Microphotograph of (a) the haptoral structures and (b) the male copulatory organ and vagina.**

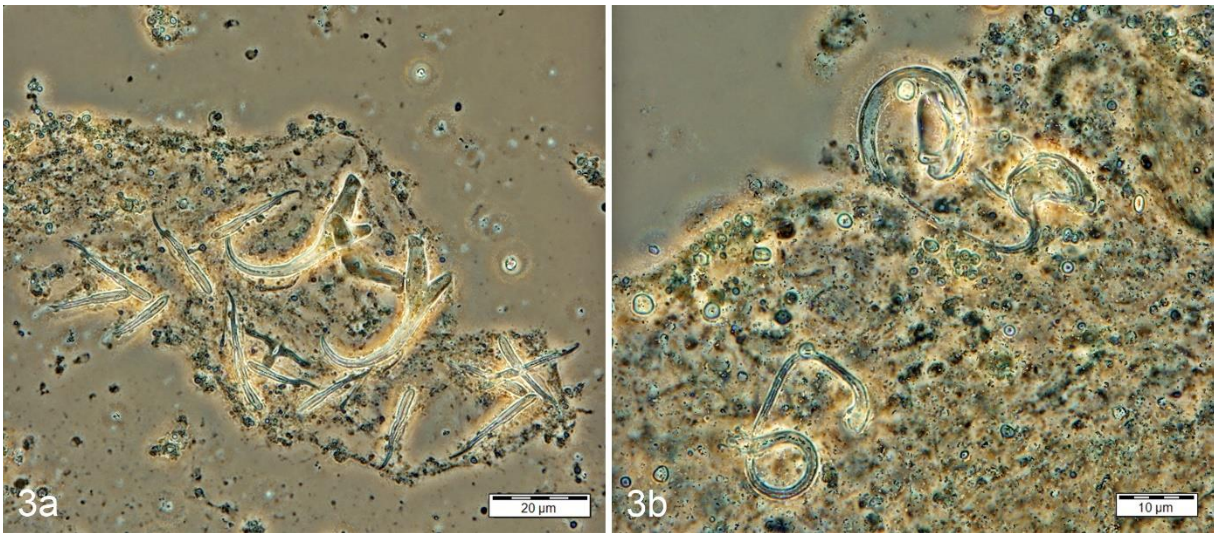

Fig. S4 ***Dactylogyrus rivalis*** **n. sp. ex** ***Squalius lepidus*****. Microphotograph of (a) the haptoral structures and (b) the male copulatory organ and vagina.**

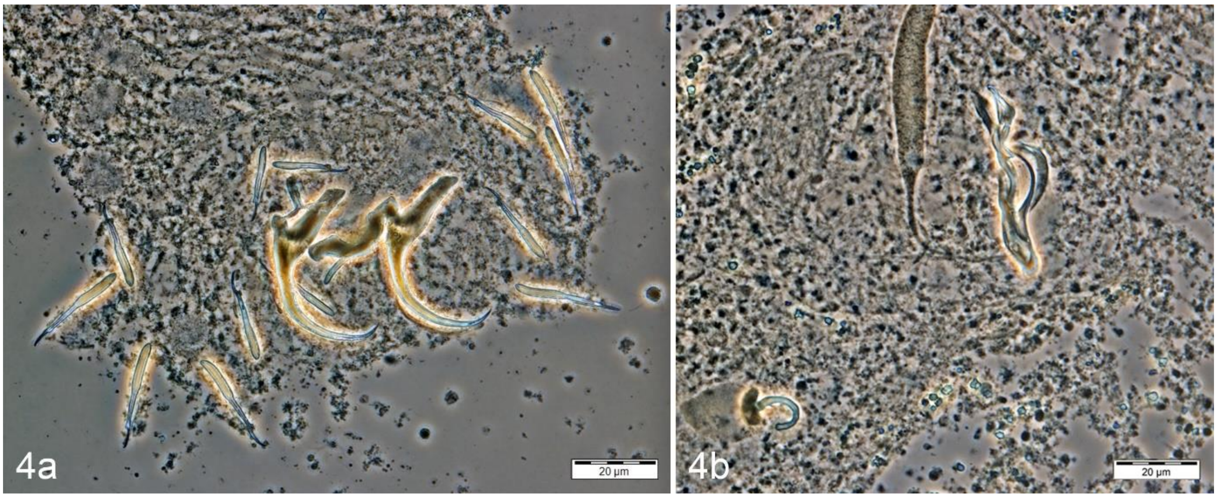

Fig. S5 Microphotographs of the newly-described *Gyrodactylus* spp.
(A) photo illustrating haptor of *G.* a*zeezsaeedi* n. sp. ex. *Squalius berak*;
(B) photo illustrating haptor of *G. blazeki* n. sp. ex *Alburnus* sp.;
(C) photo illustrating haptor of *G. iraqemembranatus* n. sp. ex *Paracapoeta trutta*;
(D) photo illustrating haptor of *G. jurajdai* n. sp. ex *Chondrostoma regium*;
(E) photo illustrating haptor of *G. mhaiseni* n. sp. ex *Alburnus sellal*;
(F) photo illustrating haptor of *G. sandai* n. sp. ex *Capoeta umbla*;
(G) photo illustrating haptor of *G. satanicus* n. sp. ex *Garra rufa*;
(H) photo illustrating haptor of *G. vukicae* n. sp. ex *Garra rufa*

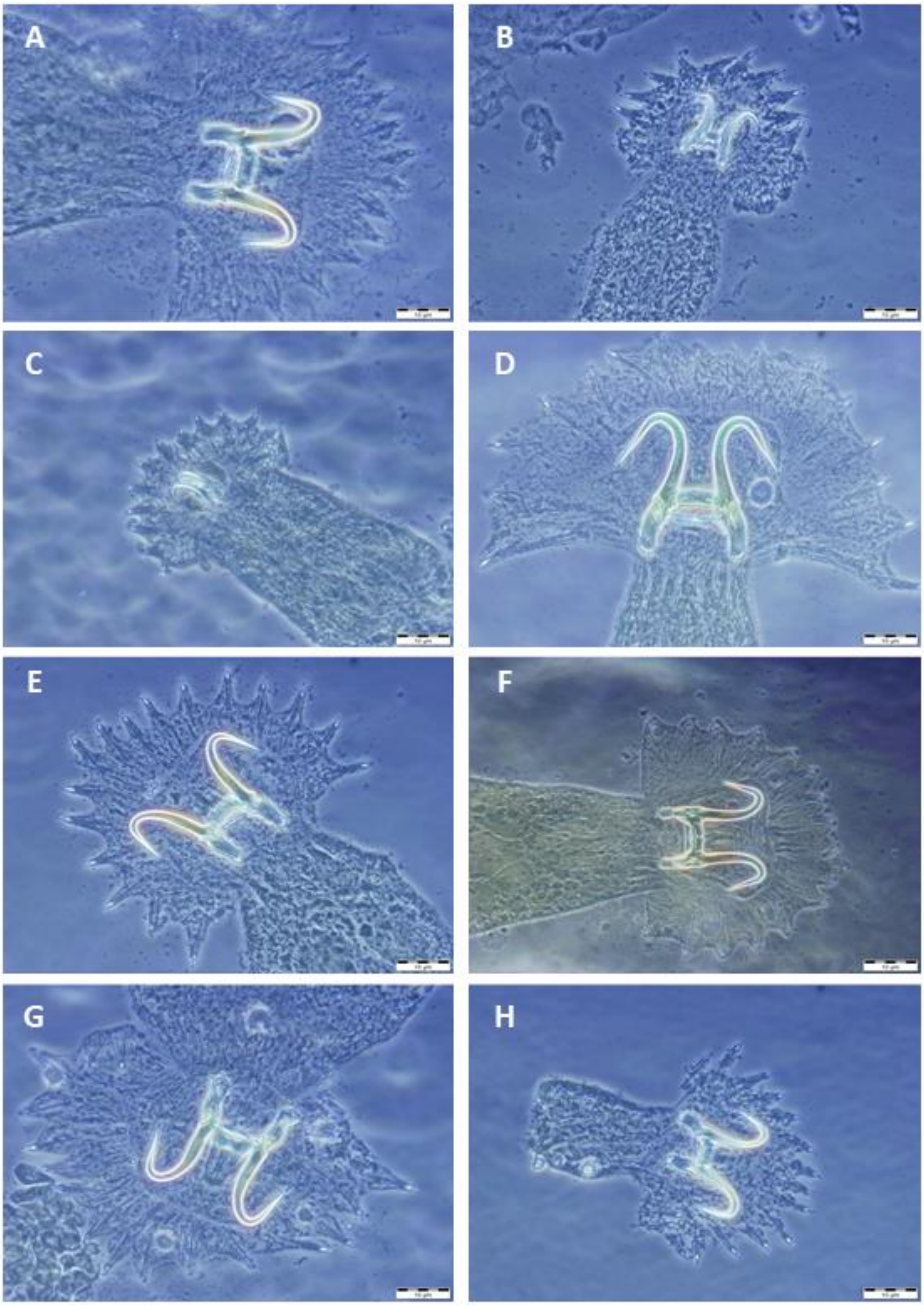

Fig. S6 Microphotographs of the collected Dogielius spp.
(A) photo illustrating haptor+MCO+Vagina of *Dogielius molnari* ex. *Cyprinion macrostomum*;
(B) photo illustrating the haptor of *D. mokhayeri* ex. *Paracapoeta trutta*;
(C) photo illustrating MCO+Vagina of *D. mokhayeri* ex. *Paracapoeta trutta*;
(D) photo illustrating the haptor of *Dogielius cf*. *persicus* ex. *Carasobarbus luteus*;
(E) photo illustrating MCO+Vagina of *Dogielius cf*. *persicus* ex. *Carasobarbus luteus*

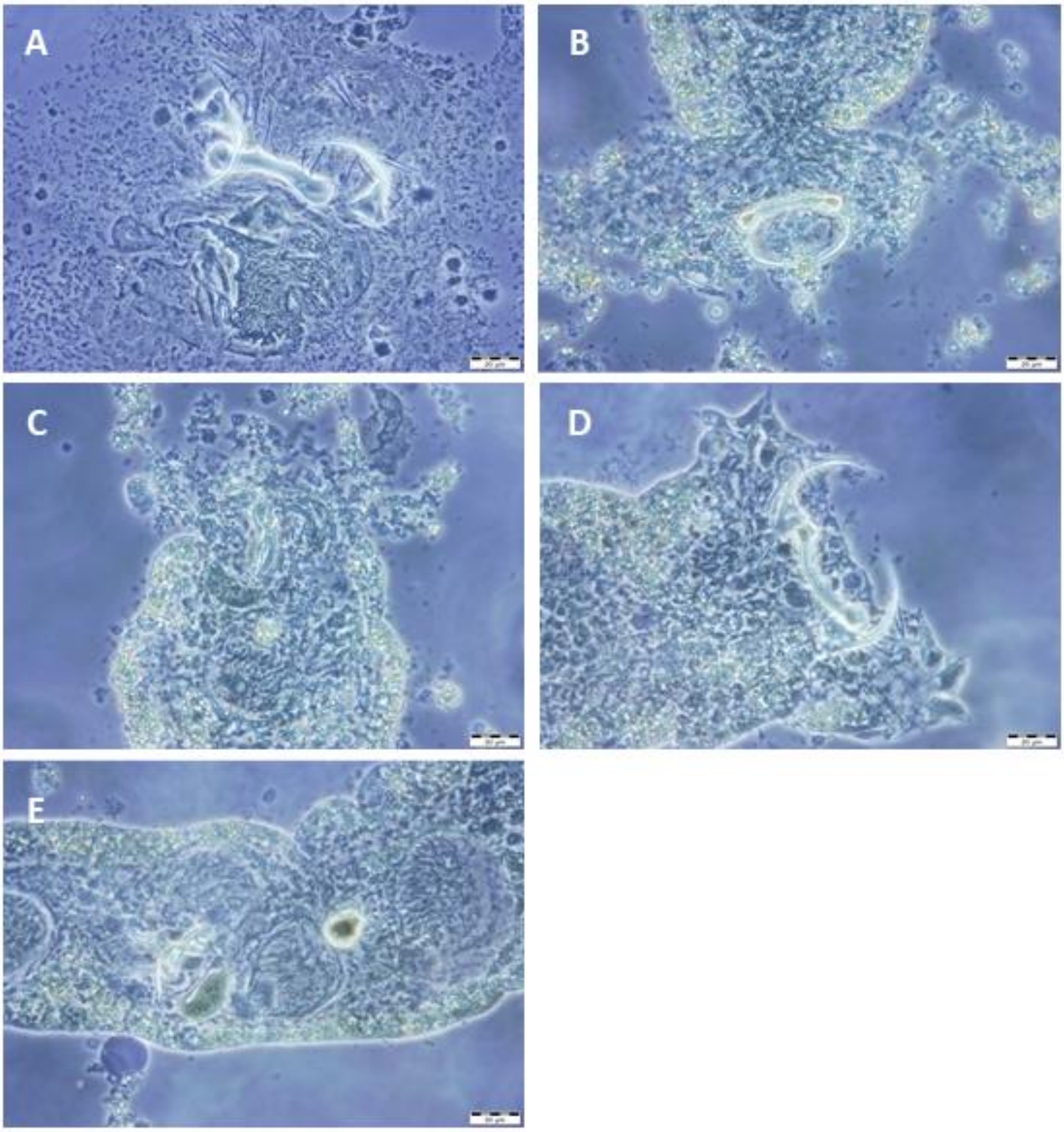

Supplement: Benovics et al. supplementary material 2 — Benovics et al. supplementary material [file S0031182023001348sup002.pdf]
